# Evaluation of LPS-Induced Acute Lung Injury Attenuation in Rats by Aminothiazole-Paeonol Derivatives

**DOI:** 10.3390/molecules22101605

**Published:** 2017-09-25

**Authors:** Pin-Kuei Fu, Chi-Yu Yang, Su-Chin Huang, Yu-Wen Hung, Kee-Ching Jeng, Ying-Pei Huang, Hong Chuang, Nai-Chun Huang, Jui-Ping Li, Ming-Hua Hsu, Jen-Kun Chen

**Affiliations:** 1 Department of Critical Care Medicine, Taichung Veterans General Hospital, Taichung 40705, Taiwan; yetquen@gmail.com; 2 Department of Biotechnology, Hungkuang University, Taichung 43302, Taiwan; 3 School of Chinese Medicine, China Medical University, Taichung 40447, Taiwan; 4 Animal Technology Laboratory, Agriculture Technology Research Institute, Miaoli 35053, Taiwan; chiyu@mail.atri.org.tw (C.-Y.Y.); pp33887788@gmail.com (Y.-W.H.); 5 Institute of Biomedical Engineering and Nanomedicine, National Health Research Institutes, Miaoli 35053, Taiwan; chin@nhri.org.tw (S.-C.H.); anai@nhri.org.tw (N.-C.H.); piny72@nhri.org.tw (J.-P.L.); 6 Department of Medical Research, Tungs’ Taichung MetroHarbor Hospital, Taichung 43503, Taiwan; kcjeng@gmail.com; 7 Department of Chemistry, National Tsing Hua University, Hsinchu 30013, Taiwan; suzann800217@hotmail.com (Y.-P.H.); hom770706@gmail.com (H.C.); 8 Department of Chemistry, National Changhua University of Education, Changhua County 50007, Taiwan; minghuahsu@cc.ncue.edu.tw; 9 Graduate Institute of Life Sciences, National Defense Medical Center, Taipei 11490, Taiwan; 10 School of Dentistry, National Defense Medical Center, Taipei 11490, Taiwan

**Keywords:** paeonol, aminothiazole-paeonol derivatives, *Moutan Cortex Radicis*, anti-inflammation agents, acute lung injury, acute respiratory distress syndrome

## Abstract

Paeonol is a key phenolic compound in the root bark of *Moutan Cortex Radicis* that has been used in traditional Chinese Medicine to ameliorate inflammation. A series of aminothiazole-paeonol derivatives (APDs) were synthesized in this work and subjected to preliminary evaluation in cells followed by verification in animals. Quantification of monocyte chemotactic protein-1 (MCP-1) and interleukin-6 (IL-6) in culture media of LPS-activated A549 cells, a lung epithelial adenocarcinoma cell line, were used to investigate the anti-inflammatory capability of APDs. ALI-bearing rats were employed to verify therapeutic efficacy of APDs according to observations of total cells, protein amounts, MCP-1 and IL-6 in bronchoalveolar lavage fluid (BALF). Histopathological examinations of lung tissues were consequently applied for validation of APDs. Among these compounds, 2-(2-aminothiazol-4-yl)-5-methoxyphenol (**4**) had the most potent activity, showing comparable inhibition of MCP-1/IL-6 and superior elimination of neutrophil infiltration and protein exudation in lungs compared to others as well as dexamethasone. This study demonstrated a comprehensive strategy to evaluate APDs through integration of cell-based screening and animal-based verification. In order to fulfill unmet needs of treating acute lung injury (ALI) and acute respiratory distress syndrome (ARDS), APDs introduced in this work could be promising lead compounds to develop high potent anti-inflammation agents.

## 1. Introduction

Herbal medicine and plant-derived compounds have been used to alleviate inflammatory diseases [1,2,3]. Paeonol is a representative phenolic compound found in the root bark of *Moutan Cortex Radicis* (MCR), reported to inhibit proinflammatory cytokines, cell surface adhesion molecules, and reactive oxygen species (ROS) [4,5,6,7,8]. Both MCR and purified paeonol were capable of attenuating LPS-induced ALI in rats by eliminating expression of myeloperoxidase (MPO) activity, iNOS, interleukin-1β (IL-1β), interleukin-6 (IL-6) and macrophage-inflammatory peptide-2 (MIP-2) [9,10]. Recently, paeonol derivatives have been synthesized and assessed for their potential anti-atherogenic, anti-cancer, anti-oxidant, and anti-virus properties [11,12,13,14]. The 2-aminothiazole moiety not only serves as a stable bioisostere of phenol groups but provides lipophilicity to improve oral availability [15]. Indeed, the 2-aminothiazole moiety has been identified to be an effective pharmacophore for different therapeutic purposes [16,17].

In the present work, we coupled 2-aminothiazole moieties with paeonol to prepare aminothiazole-paeonol derivatives (APDs) in order to treat acute lung injury (ALI) and acute respiratory distress syndrome (ARDS). The ALI and ARDS present severe inflammatory cascades and in conjunction with injury in the respiratory tract, are major health concerns that lead to high mortality and morbidity in critically ill patients [18,19,20]. Many clinical trials using drugs such as aspirin, β_2_-agonists, omega-3 fatty acids, prostaglandin E1, glucocorticoids, *N*-acetylcysteine and statins, were frustrated due to poor results whereby these treatments neither prevented nor healed ALI/ARDS [21,22,23].

Since ALI/ARDS is a complex disease associated with many biological responses, it is important to utilize not only in vitro experiments in cell lines but to also verify the results seen in animal models in order to better evaluate the effect of the most potent APDs. Several animal models, for example in vivo intratracheal administration of LPS [24,25,26], are clinically relevant and have been successfully used in previous studies. Neutrophil extracellular traps (NETs) accompanied by disruption of epithelial integrity and leakage of protein and fluid in the lungs are commonly observed in both patients and LPS-induced ALI animal models [27,28,29]. Migration and accumulation of neutrophils in airspaces are associated with up-regulation of adhesion molecules, chemotaxis, and proinflammatory cytokines in ALI and ARDS [29,30,31]. Various pro-inflammatory cytokines, including IL-1β, IL-6 and tumor necrosis factor (TNF-α), were involved in the recruitment of neutrophils to promote inflammation. 

The levels of pro-inflammatory cytokines are strongly correlated with survival in ALI/ARDS patients [32,33]. A previous study indicated monocyte chemoattractant protein (MCP)-1 was an activating factor in the LPS-induced ALI animal model [34]. The MCP-1 regulated through the JNK signaling pathway might participate in lung injury by modulating adhesion of inflammatory cells and activation of monocytes [35,36]. Yong et al. suggested using plasma MCP-1 to predict the severity of patients with pneumonia [37]. Chen et al. have demonstrated the MCP-1 in BALF could be used as a critical marker for pulmonary inflammation [38]. The MCP-1 was also employed to evaluate bleomycin-induced lung injury [39]. As for IL-6, Liang et al. have reported IL-6 could promote type 2 alveolar epithelial cells (AEC2) renewal [40]. Therefore, we aimed to use MCP-1 and IL-6 as preliminary markers for cell-based screening to evaluate anti-inflammation potency.

In this study, LPS-activated A549 cells for preliminary screening could be integrated with LPS-induced ALI-bearing rats for verification. Changes of MCP-1 and IL-6 in culture media of LPS-activated A549 cells were employed to evaluate the anti-inflammatory activities of APDs compared to reference compounds (paeonol and dexamethasone). Furthermore, relatively high potent APDs were subjected to verification in ALI-bearing rats. Comprehensive investigation of cell infiltration, protein accumulation and inflammatory markers secretion in both of bronchoalveolar lavage fluid (BALF) and lung tissues demonstrate an effective strategy to explore optimal APDs for the therapy of ALI/ARDS.

## 2. Results

### 2.1. Synthesis

**Scheme 1:**
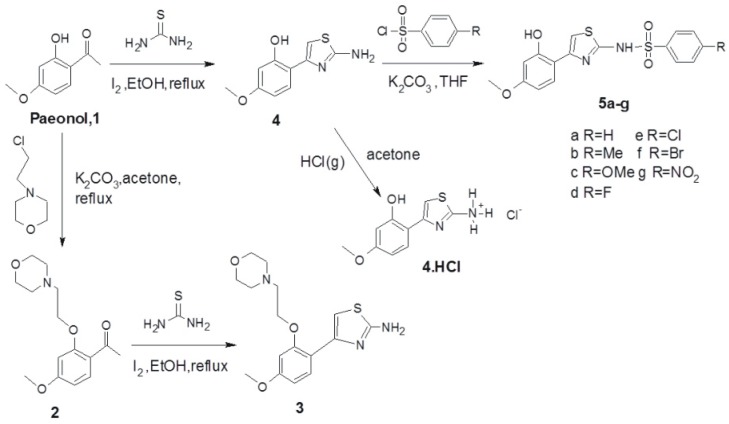
Synthesis of aminothiazole-paeonol derivatives (APDs) as anti-inflammation agents in this study.

The preparation of the vaminothiazole-paeonol derivatives is shown in Scheme 1. Paeonol (**1**) was treated with 2-chloroethylmorpholine to generate morpholine-*o*-paeonol (**2**). Compound **2** was reacted with thiourea and iodine to obtain morpholine-*o*-paeonol aminothiazole (**3**). Additionally, paeonol (**1**) could be treated with thiourea and iodine to construct the 2-aminothiazole scaffold **4** through the condensation–cyclization of thiourea. Compound **4** was reacted with excess hydrochloric acid gas to generate the aminothiazole-paeonol salt **4.HCl**. Paeonol-2-aminothiazole-phenylsulfonyl derivatives **5a**–**5g** were obtained reacting compound **4** with substituted phenylsulfonyl chlorides. These products were obtained in sufficient yields and purified by using recrystallization for the subsequent cell-based and animal-based experiments. 

### 2.2. MCP-1 and IL-6 Secretion in LPS-Activated A549 Cells

**Figure 1:**
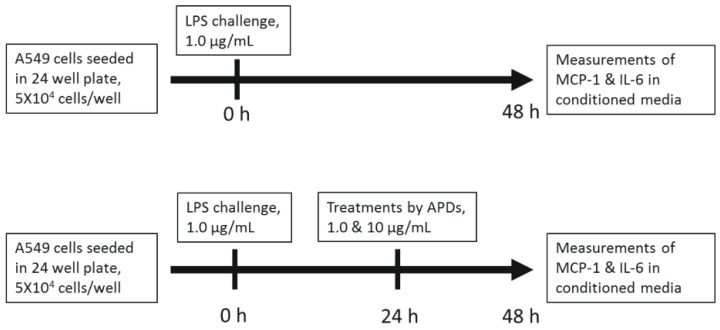
Preliminary screening protocols for anti-inflammation potency of APDs using the LPS-activated A549 cell model.

**Figure 2:**
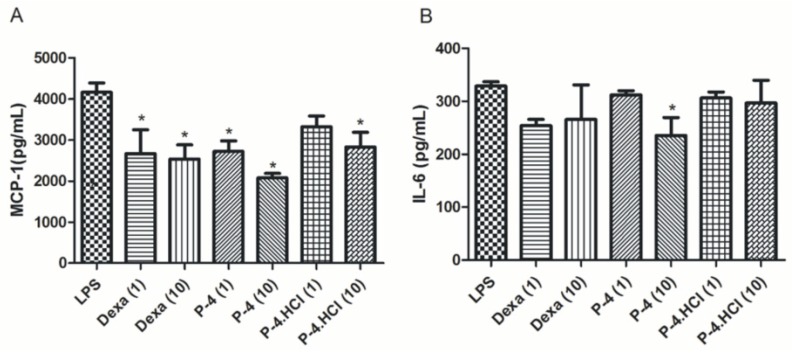
Secretion of (**A**) MCP-1 and (**B**) IL-6 in conditioned media of LPS-activated A549 cells treated with Dexa (1.0 µg/mL and 10 µg/mL) and selected APDs (1.0 µg/mL and 10 µg/mL). Significant differences between each treatment group and LPS-activated (positive control) group at *p* values less than 0.05 were annotated with *.

Low and high concentrations of paeonol, dexamethasone (Dexa) and APDs (1.0 µg/mL and 10 µg/mL) were used to treat LPS-activated A549 cells following the protocols shown in Figure 1. A549 is a lung epithelial adenocarcinoma cell line. The MCP-1 in conditioned media was quantified to survey anti-inflammatory capability of APDs. Secretion of MCP-1 was significantly inhibited by compound **4** and Dexa at 1.0 µg/mL and 10 µg/mL (Table 1 and Figure 2A). The compound **4.HCl** at 10 µg/mL could inhibit of MCP-1 secretion (Figure 2A). Only compound **4** at 10 µg/mL suppressed IL-6 secretion but others didn't at neither high nor low concentrations (Figure 2B).

### 2.3. Cell Viabilities for APDs Treatments

**Figure 3:**
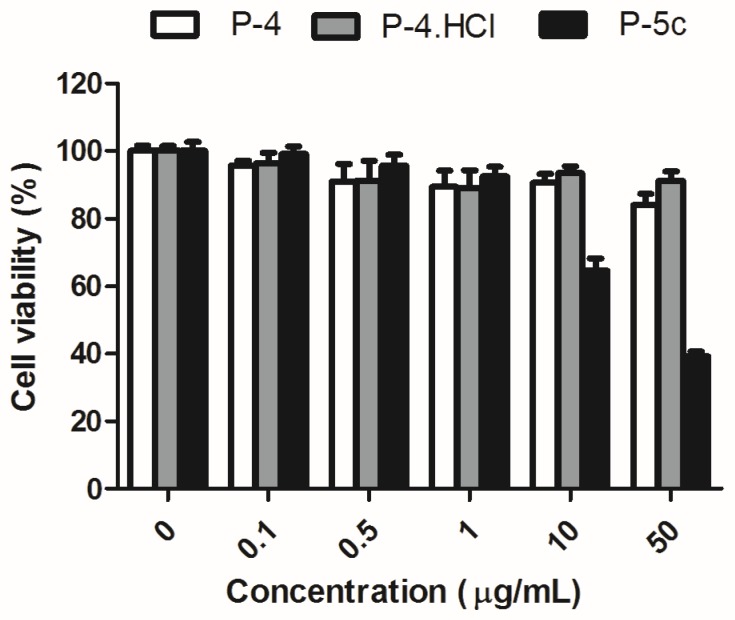
Viabilities of A549 cells at 24 h post-treatment of selected APDs. The P-4, P-4.HCl and P-5c represented the Compound **4**, **4.HCl** and **5c** shown in Figure 1.

Cell viabilities for APDs against A549 cells were determined by WST-1 assays. Compound **4** and compound **4.HCl** are very biocompatible because of more than 90% cell survive a 50 µg/mL treatment (Figure 3). Compound **5c**, showing an IC_50_ value of 39 µg/mL (127 µM) is the most cytotoxic APD prepared in this study.

### 2.4. Comparison of BALF Collected from ALI-Bearing Rats

**Figure 4:**
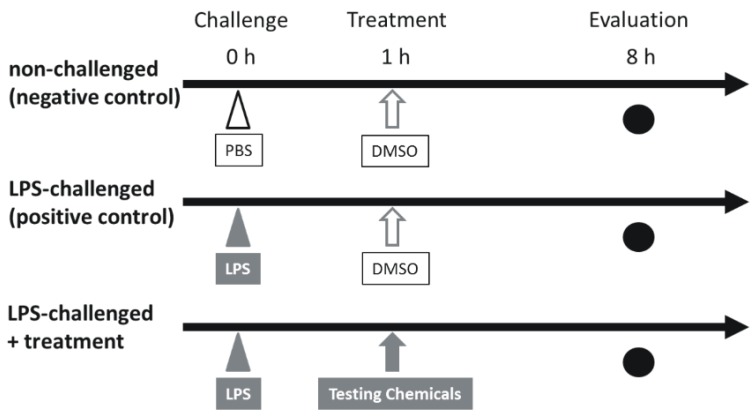
Verification protocols of LPS-challenged ALI-bearing rats treated with testing chemicals (APDs, paeonol, and dexamethasone).

Compound **4** and Compound **4.HCl** are promising APDs based on our preliminary screening in a LPS-activated A549 cell model. To further verify therapeutic efficacy, paeonol, compound **4**, compound **4.HCl** and Dexa were injected i.p. into the ALI-bearing rat model following the protocols shown in Figure 4. 

**Figure 5:**
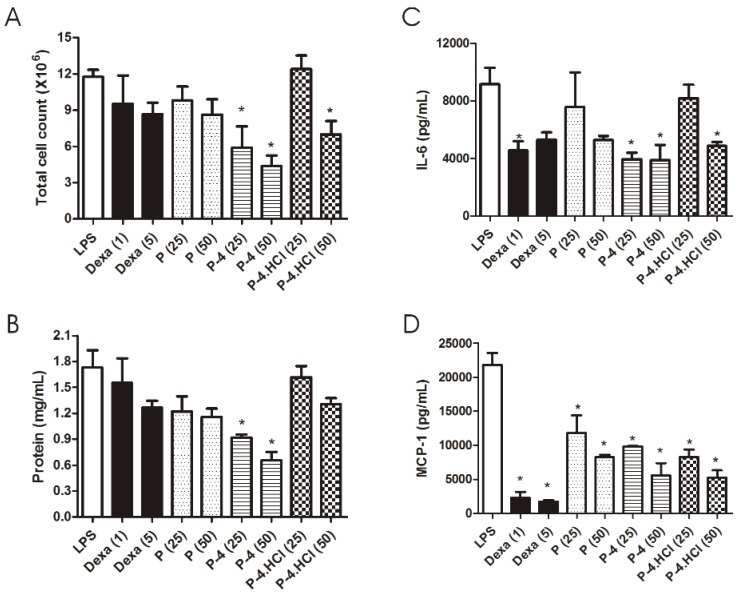
Biological responses of ALI-bearing rats treated with Dexa, paeonol and selected APDs on (**A**) total cell counts, (**B**) total protein concentrations, (**C**) IL-6 and (**D**) MCP-1 in bronchoalveolar lavage fluid (BALF). Significant differences between each treatment group and LPS-treated (positive control) group at *p* values less than 0.05 were annotated with *.

Previous studies have indicated both of endotoxin-induced ALI animal models and endotoxin-treated human showed time-dependent changes in either peripheral inflammatory responses or accumulation of immune cells, total proteins, and cytokines in BALF associated with pathological lung injury within 48 h after endotoxin-challenge [41,42]. The severity peak was observed in ALI-bearing rats at 8 h after LPS challenge [43]. Therefore, changes of total cell counts, cytokines (IL-6 and MCP-1) and total proteins in BALF were determined in order to validate therapeutic efficacy of APDs (Figure 5). 

Paeonol and Dexa proved ineffective at inhibiting cell infiltration in lungs of ALI-bearing rats (Figure 5A). Notably, compound **4** eliminated total cell counts in BALF at both 25 mg/kg and 50 mg/kg treatment. As for compound **4.HCl**, effective inhibition of cell infiltration in lungs was observed at 50 mg/kg treatment. Compound **4** was thus superior to paeonol, Dexa, and compound **4.HCl** and substantially inhibited protein infiltration in lungs of ALI-bearing rats (Figure 5B). Paeonol (50 mg/kg), compound **4** (25 and 50 mg/kg), compound **4.HCl** (50 mg/kg) and Dexa (1 and 5 mg/kg) suppressed IL-6 secretion in BALF (Figure 5C), in which compound **4** and Dexa presented comparable efficacies for the two dosages. The compound **4.HCl** and paeonol presented comparable efficacies merely at 50 mg/kg. Paeonol, **4**, and **4.HCl** were comparable at inhibiting MCP-1 secretion at both of low and high dosages (Figure 5D). 

### 2.5. Comparison of Compound ***4*** and Compound ***4.HCl*** by Histopathological Examinations

**Figure 6:**
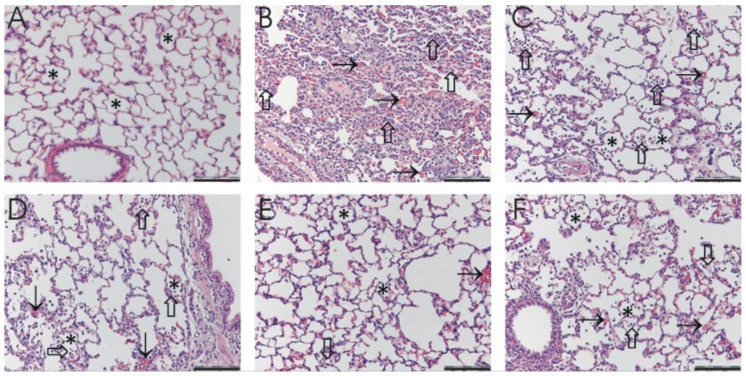
Histopathological examination of H&E stained lung tissues collected from: (**A**) non-challenged (negative control), (**B**) LPS-challenged (positive control) rats without treatment and ALI-bearing rats treated with (**C**) Dexa, (**D**) paeonol, (**E**) Compound **4**, and (**F**) Compound **4.HCl**. Rats were consequently sacrificed at 8 h after intratracheal administration of LPS (8 mg/kg). The alveolar spaces (∗) filled with the mixture of red blood cells (→) and mononuclear/neutrophilic infiltrates (⇧) combined with biological responses observed in BALF were integrated to comprehensively evaluate therapeutic efficacy of APDs. (Scale bar: 100 µm).

Lung tissues for histopathological examination were collected at 8 h after LPS administration. No prominent neutrophil infiltration, red blood cells and protein exudates were observed in the negative control group (Figure 6A). ALI-bearing rats presented extremely severe protein exudation and cells infiltration in lungs (Figure 6B), which could be eliminated after treatment with Dexa (Figure 6C), paeonol (Figure 6D), compound **4** (Figure 6E) and compound **4.HCl** (Figure 6F). Compound **4** is superior to Dexa, paeonol and compound **4.HCl** at alleviating LPS-induced acute lung injury.

## 3. Discussion

Herbal medicines are invaluable resources to construct plant-derived compound libraries for preventing and treating diseases. For example, paeonol (**1**, Scheme 1) is a one of the remarkable molecules in *Moutan Cortex Radicis* (MCR) that has been used in traditional Chinese medicine to promote blood circulation and eliminate blood stasis [44,45]. Our previous work described an animal model treated with MCR to alleviate ALI through inhibiting MCP-1 and IL-8 secretion [10]. In addition to MCP-1 and IL-8, elimination of TNF-α, IL-1β, IL-6, and PAI-1 in BALF were observed in ALI-bearing rats that were treated with paeonol purified from MCR [9]. Paeonol derivatives may also have extensive biomedical applications. Zhu et al. reported thiosemicarbazone paeonol derivatives were potential inhibitors of mushroom tyrosinase [46]. Tsai et al. recently demonstrated a series of aminothiazole paeonol derivatives (APDs) as anticancer agents with relatively high potency against gastrointestinal adenocarcinoma (AGS and HT-29 cells) [14]. The thiazole ring of APDs may contribute to a variety of biological effects, which encourage us to evaluate anti-inflammatory responses for a series of APDs. 

ALI and ARDS present acute respiratory failure with severe inflammation in the respiratory tract, leading to high mortality in critically ill patients [18,47]. Inflammation disrupting lung epithelial and endothelial barriers cause damage of alveolar-capillary membrane integrity, excessive trans-epithelial neutrophil migration, and release of pro-inflammatory/cytotoxic mediators. Many therapeutics involved in different underlying mechanisms have been applied to treat ALI/ARDS; however, most of them rarely reduce mortality [21,48,49].

In this work, we have established a workflow consisting of primary cell-based screening (Figure 1) followed by animal-based verification (Figure 4) to propel evaluation of potential APDs against ALI. Figure 3 presents MCP-1 rather than IL-6 in conditioned media is a relatively sensitive marker to monitor how LPS-activated A549 cells responsive to APDs treatments because concentration of MCP-1 is approximately 10-fold higher than that of IL-6 in conditioned media. The MCP-1 in conditioned media of negative control group (without challenge of LPS) was 1411 pg/mL versus 4164 pg/mL in that of LPS-activated group (positive control), showing a 2.95-fold increase of MCP-1 caused by LPS challenge. On the other hand, IL-6 in conditioned medium showed a 2.42-fold increase (from 136 pg/mL to 329 pg/mL) caused by LPS challenge. 2-(2-Aminothiazol-4-yl)-5-methoxyphenol (**4)** and 2-(2-aminothiazol-4-yl)-5-methoxyphenol hydrochloride (**4.HCl**) significantly suppressed MCP-1 secretion (Figure 2A) and showed comparable efficacy to Dexa, a synthetic corticosteroid for treating inflammatory and autoimmune symptoms. Figure 2B shows that 10 µg/mL of compound **4** could effectively inhibit IL-6 secretion in culture media of LPS-activated A549 cells. Both compound **4** and compound **4.HCl** are superior to compound **5c** because of very mild cytotoxicity shown in Figure 3, which suggests compound **4** and compound **4.HCl** could be safely used for anti-inflammatory medications for ALI/ARDS.

Compound **4** and compound **4.HCl** were subsequently verified in ALI-bearing rats. The compound **4** is excellent at reducing cell infiltration and protein exudation in the alveolar space and simultaneously inhibits secretion of MCP-1 and IL-6 in BALF (Figure 5). However, compound **4.HCl** shows limited effects (effective at the high dose: 50 mg/kg) in reducing cell infiltration (Figure 5A) and IL-6 secretion in lungs (Figure 5C) despite effective elimination of MCP-1 (Figure 5D) compared to compound **4**. In comparison with compound **4** and compound **4.HCl**, Dexa presents very effective elimination of IL-6 and MCP-1 in BALF (Figure 5C,D); but Dexa is less responsive for reduction of cell infiltration and protein exudation (Figure 5A,B).

To further validate therapeutic efficacy of compounds **4** and **4.HCl**, histopathological examination of lung tissues (Figure 6) was employed to identify optimal APDs for treating ALI/ARDS. Infiltration of neutrophils, exudation of red blood cells and protein were extremely severe in ALI-bearing rats (Figure 6B) compared with non-challenged rats (Figure 6A), as determined by the extensive presence of cellular debris and proteinaceous material in lung tissues. The remarkable characteristic of ALI/ARDS is neutrophils recruited from the peripheral blood to the alveoli during active infections [50,51,52], which was simultaneously observed in ALI-bearing rats (Figure 6B). Neutrophil infiltration into the lungs is mediated through a complex network of chemokines/cytokines secreted by alveolar macrophages. Chemokines and cytokines including TNF-α, IL-1β, IL-6, IL-8, IL-10, and MCP-1 can promote chemotaxis and recruit more neutrophils into injured lungs [4,18,53,54,55,56]. Infiltration of neutrophils and red blood cells associated with protein exudates are alleviated to different levels by treating either Dexa (Figure 6C), paeonol (Figure 6D), compound **4** (Figure 6E) or compound **4.HCl** (Figure 6F). Despite the dramatic inhibition of MCP-1 caused by Dexa (Figure 5D), it does not effectively eliminate protein exudation (Figure 5B) and neutrophil infiltration, which is again proved by the histopathological examination (Figure 6C). Treatment of compound **4** (Figure 6E) presents effective response to eliminate neutrophil infiltration and protein exudation compared to treatments of compound **1** (Figure 6D) and compound **4.HCl** (Figure 6F). We therefore suggest the compound **4** is highly efficacious for treating ALI/ARDS because of its comprehensive activity to eliminate neutrophil infiltration, protein exudation and chemokine/ cytokine secretion.

## 4. Materials and Methods 

### 4.1. Chemicals and General Procedures

Solvents, including dichloromethane, ethanol, ethyl acetate, hexane, methanol and tetrahydrofuran were purchased from Mallinckrodt Pharmaceuticals (St. Louis, MO, USA). Ethyl acetate was dried and distilled from CaH_2_. Tetrahydrofuran was dried by distillation from sodium and benzophenone under an atmosphere of nitrogen. Benzenesulfonyl chloride, 4-bromobenzenesulfonyl chloride, 4-chlorobenzenesulfonyl chloride, 4-fluorobenzenesulfonyl chloride, 4-methoxybenzene-sulfonyl chloride, 4-nitrobenzenesulfonyl chloride, potassium carbonate, and *p*-toluenesulfonyl chloride were purchased from Sigma-Aldrich (St. Louis, MO, USA). All reactions were carried out in oven-dried glassware (120 °C) under an atmosphere of nitrogen unless as indicated otherwise. The lipopolysaccharide (LPS) from *E. coli* (serotype O55:B5) was purchased from Sigma-Aldrich. Dexamethasone-21-phosphate disodium (Dexa) was purchased from Standard Chem. & Pharm. Co., Ltd. (Tainan, Taiwan). 

Analytical thin layer chromatography was performed on pre-coated plates (silica gel 60 F-254), purchased from Merck (Merck KGaA, Darmstadt, Germany). Proton (^1^H)-NMR spectra were obtained on Mercury-400 (400 MHz) (Varian, Palo Alto, CA, USA) AC-400 (400 MHz) (Bruker Biospin GmbH, Rheinstetten, Germany) or Avance 500 (500 MHz) (Bruker) spectrometers using chloroform-*d* (CDCl_3_), dimethylsulfoxide-*d_6_* (DMSO-*d*_6_) and deuterium oxide (D_2_O) as the solvents. Chemical shifts of ^1^H-NMR were referenced to residual protonated solvents (δ 7.24 for chloroform, δ 2.49 for dimethylsulfoxide and 4.79 for deuterium oxide). Carbon-13 NMR spectra were obtained on a Varian Mercury-400 (100 MHz) or Bruker Avance 500 (125 MHz) spectrometer using chloroform-*d* (CDCl_3_), dimethylsulfoxide-*d*_6_ (DMSO-*d*_6_) and methanol-d_4_ (CD_3_OD) as the solvents. Chemical shifts of ^13^C-NMR were referenced to the center of the CDCl_3_ triplet (δ 77.0 ppm), DMSO septet (δ 39.5 ppm) and methanol septet (δ 49.0 ppm). Multiplicities were recorded by following abbreviations: s, singlet; d, doublet; t, triplet; q, quartet; m, multiplet; *J*, coupling constant (hertz). NMR spectra are presented in the Supplementary Materials (Figures S1–S20). High-resolution mass spectra were obtained by a JMS-700 mass spectrometer (JEOL Ltd., Tokyo, Japan). 

### 4.2. Procedure for the Preparation of Morpholine-O-Paeonol (***2***)

A solution containing paeonol (**1**, 1.0 equiv.), 2-chloroethylmorpholine (1.2 equiv.), potassium carbonate (3.0 equiv.) and ammonium chloride (1.5 equiv.) in acetone (30.0 mL) was refluxed for 12 h. Then the mixture was extracted with dichloromethane for three times, and dried over MgSO_4(s)_. After concentration of the solvent, the residue was purified by column chromatography over silica gel using methanol and dichloromethane as eluent to afford *1-(4-methoxy-2-(2-morpholinoethoxy)- phenyl)ethanone* (**2**) as a white solid. Yield 90%; ^1^H-NMR (500 MHz, CDCl_3_): δ = 7.81 (d, *J* = 8.5 Hz, 1H), 6.51 (dd, *J* = 2, 8.5 Hz, 1H), 6.42 (d, *J* = 2 Hz, 1H), 4.14 (t, *J* = 5.5 Hz, 2H), 3.83 (s, 3H), 3.70 (t, *J* = 4.5 Hz, 4H), 2.83 (t, *J* = 5.5 Hz, 2H), 2.54 (t, *J* = 4.5 Hz, 4H); ^13^C-NMR (125 MHz, CDCl_3_): δ = 197.5, 164.3, 160.0, 132.5, 121.2, 105.3, 98.9, 66.7, 65.9, 57.2, 55.4, 53.8, 31.9; HRMS (ESI^+^): *m/z* [M]^+^ calc. for C_15_H_21_NO_4_ 279.1471, found 280.1578 for [M + H]^+^.

### 4.3. Procedure for the Preparation of Morpholine-O-Paeonol Aminothiazole (***3***)

To obtain aminothiazole paeonol **3**, compound **2** (1.0 equiv) was reacted with iodine (1.1 equiv) and thiourea (3.0 equiv) in ethanol under reflux condition for 12–16 h. Then, the mixture was quenched with NaOH(aq) (2.0 equiv) and the ethanol was removed under reduced pressure. The residue was extracted with ethyl acetate and the combined organic layer was washed brine and dried over MgSO_4(s)_. After being filtered and condensed under reduced pressure, the crude product was purified by column chromatography on silica gel (ethyl acetate and hexane as eluent) to obtain compound *4-(4-methoxy-2-(2-morpholinoethoxy)phenyl)thiazol-2-amine* (**3**) as a whit solid. Yield 49%; ^1^H-NMR (500 MHz, DMSO-*d*_6_): δ = 7.93 (d, *J* = 10 Hz, 1H), 7.30 (s, 1H), 6.85 (s, 2H), 6.62 (s, 1H), 6.54 (d, *J* = 10 Hz, 1H), 4.15 (s, 1H), 3.76 (s, 3H), 3.57 (s, 4H), 2.76 (s, 2H); ^13^C-NMR (125 MHz, DMSO-*d_6_*): δ = 166.0, 159.2, 156.8, 145.7, 129.9, 116.7 105.1, 103.7, 99.4, 66.3, 65.1, 56.9, 55.2, 53.3; HRMS (ESI^+^): *m/z* [M]^+^ calc. for C_16_H_21_N_3_O_3_S 335.1298, found 336.1375 for [M + H]^+^.

### 4.4. Procedure for the Preparation of Aminothiazole-Paeonol (***4***)

To obtain aminothiazole-paeonol (**4**), paeonol (**1**, 1.0 equiv) was reacted with iodine (1.1 equiv) and thiourea (3.0 equiv) in ethanol under reflux condition for 12–16 h. Then, the mixture was quenched with NaOH_(aq)_ (2.0 equiv) and the ethanol was removed under reduced pressure. The residue was extracted with ethyl acetate and the combined organic layer were washed brine and dried over MgSO_4(s)_. After being filtered and condensed under reduced pressure, the crude product was purified by column chromatography on silica gel (ethyl acetate and hexane as eluent) to give compound *2-(2-aminothiazol-5-yl)-5-methoxyphenol* (**4**). ^1^H-NMR (400 MHz, CDCl_3_): δ 7.40 (d, *J* = 8.4 Hz, 1H, H-3), 6.54 (s, 1H, CH), 6.47 (s, 1H, H-6), 6.42 (dd, *J* = 8.4, 2.0 Hz, 1H, H-4), 5.05 (s, 2H, NH_2_), 3.78 (s, 3H, OMe) ppm. ^13^C-NMR (100 MHz, CDCl_3_): δ 166.8, 161.0, 157.3, 148.9, 126.6, 111.0, 106.8, 101.6, 98.9, 55.2 (OMe) ppm; HRMS (ESI^+^): *m/z* [M]^+^ calc. for C_10_H_10_N_2_O_2_S_1_ 222.0458, found 223.0562 for [M + H]^+^.

### 4.5. Procedure for the Preparation of Aminothiazole-Paeonol salt (***4.HCl***)

To obtain aminothiazole-paeonol salt **4.HCl**, compound **4** (1.0 equiv) dissolved in acetone (20 mL) was reacted with excess hydrochloric acid gas that was produced by adding concentrated H_2_SO_4_ with an addition funnel to excess NaCl_(s)_. When precipitation nearly stopped, the precipitate was collected using suction filtration and washed with cold acetone to obtain the pure *4-(2-hydroxy-4-methoxyphenyl)thiazol-2-aminium chloride* (**4.HCl**) as a yellow solid. Yield 87%; ^1^H-NMR (D_2_O, 400 MHz): δ 7.37 (d, *J* = 8.4 Hz, 1H), 6.80 (s, 1H), 6.58 (dd, *J* = 6.8, 1.6 Hz, 1H), 6.48 (d, 2.0 Hz, 1H), 3.81 (s, 3H) ppm. ^13^C-NMR (CD_3_OD, 100 MHz): δ 171.4, 163.6, 157.0, 138.1, 130.1, 130.0, 109.0, 107.7, 102.5, 55.8 (OMe) ppm; HRMS (ESI^+^): *m/z* [M]^+^ calc. for C_10_H_11_ClN_2_O_2_S_1_ 258.0224, found 223.0556 for [M + H − HCl]^+^.

### 4.6. Procedures for the Preparation of Aminothiazole-Paeonol Derivatives ***5a***–***5g***

The solution containing aminothiazole-paeonol (**4**, 1.0 equiv) in anhydrous THF (2.0–3.0 mL) was added potassium carbonate (1.3 equiv) and a sulfonyl chloride (1.1 equiv). After the reaction mixture was stirred at 25 °C for 2–3 h, it was diluted with dichloromethane (5.0 mL). Inorganic solids were filtered off and the filtrate was concentrated under reduced pressure to afford the residue. It was then purified by use of column chromatography on silica gel (various ratio of methanol to dichloromethane) to give the desired conjugates **5a**–**5g**.

*N-[4-(2-Hydroxy-4-methoxyphenyl)thiazol-2-yl]benzenesulfonamide* (**5a**): Yield 80%. Green solid. IR (film): ν 3569.1, 2811.2, 1560.1, 1481.2, 1374.2, 1131.6, 853.4 cm^−1^. ^1^H-NMR (400 MHz, CDCl_3_): δ 7.62–7.52 (m, 2H, 2 × ArH), 7.43–7.31 (m, 1H, H-6), 7.28–7.25 (m, 3H, 3 × ArH), 6.98 (d, *J* = 2.0 Hz, 1H, H-3), 6.89–6.79 (m, 1H, H-5), 6.68 (s, 1H, SCH), 3.70 (s, 3H, OMe) ppm. ^13^C-NMR (100 MHz, CDCl_3_): δ 164.1, 129.7, 125.9, 113.9, 113.7, 105.0, 56.0 (OMe) ppm; HRMS (ESI^+^): *m/z* [M]^+^ calc. for C_16_H_14_N_2_O_4_S_2_ 362.0390, found 363.0396 for [M + H]^+^.

*N-[4-(2-Hydroxy-4-methoxyphenyl)thiazol-2-yl]-4-methylbenzenesulfonamide* (**5b**): Yield 84%. Off-white solid. IR (film): ν 3579.1, 2921.2, 1580.1, 1491.2, 1384.1, 1141.6, 843.4 cm^−1^. ^1^H-NMR (400 MHz, CDCl_3_): δ 7.44 (d, *J* = 8.8 Hz, 1H, H-6), 7.39 (d, *J* = 8.2 Hz, 2H, 2 × ArH), 7.06 (d, *J* = 8.2 Hz, 2H, 2 × ArH), 6.81 (d, *J* = 2.4 Hz, 1H, H-3), 6.74 (dd, *J* = 8.8, 2.4 Hz, 1H, H-5) 6.52 (s, 1H, SCH), 3.72 (s, 3H, OMe), 2.29 (s, 3H, CH_3_) ppm. ^13^C-NMR (100 MHz, CDCl_3_): δ159.3, 147.0, 145.3, 144.4, 130.5, 129.1, 128.2, 120.9, 113.1, 108.7, 105.9, 55.4 (OMe), 21.4 (CH_3_) ppm; HRMS (ESI^+^): *m/z* [M]^+^ calc. for C_17_H_16_N_2_O_4_S_2_ 376.0546, found 377.0551 for [M + H]^+^.

*N-[4-(2-Hydroxy-4-methoxyphenyl)thiazol-2-yl]-4-methoxybenzenesulfonamide* (**5c**): Yield 83%. Off-white solid. IR (film): ν 3672.1, 2931.1, 1560.6, 1473.2, 1388.1, 1142.7, 817.4 cm^−1^. ^1^H-NMR (400 MHz, CDCl_3_): δ 7.46–7.42 (m, 3H, H-6 + 2 × ArH), 6.86 (d, *J* = 2.8 Hz, 1H, H-3), 6.76–6.72 (m, 3H, H-5 + 2 × ArH), 6.57 (s, 1H, SCH), 3.75 (s, 3H, OMe), 3.74 (s, 3H, OMe) ppm. ^13^C-NMR (100 MHz, CDCl_3_): δ 166.8, 163.9, 159.4, 147.1, 144.7, 130.5, 125.9, 121.0, 113.7, 113.2, 108.8, 106.2, 55.6 (OMe), 55.5 (OMe) ppm; HRMS (ESI^+^): *m/z* [M]^+^ calc. for C_17_H_16_N_2_O_5_S_2_ 392.0495, found 393.0498 for [M + H]^+^.

*4-Fluoro-N-[4-(2-hydroxy-4-methoxyphenyl)thiazol-2-yl]benzenesulfonamide* (**5d**): Yield 81%. White solid. IR (film): ν 3695.5, 2943.3, 1589.7, 1493.9, 1378.3, 1157.7, 837.7 cm^−1^. ^1^H-NMR (400 MHz, CDCl_3_): δ 7.51–7.48 (m, 2H, 2 × ArH), 7.41 (d, *J* = 8.8 Hz, 1H, H-6), 6.95–6.91 (m, 2H, 2 × ArH), 6.86 (d, *J* = 2.6 Hz, 1H, H-3), 6.75 (dd, *J* = 8.8, 2.6 Hz, 1H, H-5), 6.46 (s, 1H, SCH), 3.74 (s, 3H, OMe) ppm. ^13^C-NMR (100 MHz, CDCl_3_): δ 167.3, 167.1, 164.5, 159.4, 146.8, 144.5, 131.2, 130.6, 120.9, 115.9, 115.6, 113.3, 108.9, 105.8, 55.5 (OMe) ppm; HRMS (ESI^+^): *m/z* [M]^+^ calc. for C_16_H_13_FN_2_O_4_S_2_ 380.0295, found 381.0302 for [M + H]^+^.

*4-Chloro-N-[4-(2-hydroxy-4-methoxyphenyl)thiazol-2-yl]benzenesulfonamide* (**5e**): Yield 81%. White solid. IR (film): ν 3708.8, 2796.7, 1566.74, 1459.55, 1314.15, 1256.54, 984.95 cm^−1^. ^1^H-NMR (400 MHz, CDCl_3_): δ 7.53 (d, *J* = 8.8 Hz, 1H, H-6), 7.48–7.46 (m, 2H, 2 × ArH), 7.29–7.27 (m, 2H, 2 × ArH), 6.94 (d, *J* = 2.4 Hz, 1H, H-3), 6.82 (dd, *J* = 8.8, 2.4 Hz, 1H, H-5), 6.56 (s, 1H, SCH), 3.81 (s, 3H, OMe) ppm. ^13^C-NMR (100 MHz, CDCl_3_): δ 167.2, 159.5, 146.8, 144.7, 140.7, 133.2, 130.7, 129.6, 128.7, 120.9, 113.5, 109.1, 106.0, 55.6 (OMe) ppm; HRMS (ESI^+^): *m/z* [M]^+^ calc. for C_16_H_13_ClN_2_O_4_S_2_ 395.9999, found 397.0076 for [M + H]^+^.

*4-Bromo-N-[4-(2-hydroxy-4-methoxyphenyl)thiazol-2-yl]benzenesulfonamide* (**5f**): Yield 83%. White solid. IR (film): ν 3691.2, 2838.7, 1591.3, 1479.1, 1362.1, 1143.7, 821.7 cm^−1^. ^1^H-NMR (400 MHz, CDCl_3_): δ 7.36–7.33 (m, 3H, H-5 + 2 × ArH), 7.28–7.26 (m, 2H, 2 × ArH), 6.80 (d, *J* = 1.6 Hz, 1H, H-3), 6.71 (dd, *J* = 8.8, 1.6 Hz, 1H, H-5), 6.35 (s, 1H, SCH), 3.69 (s, 3H, OMe) ppm. ^13^C-NMR (100 MHz, CDCl_3_): δ 166.2, 159.6, 146.9, 145.1, 133.8, 121.0, 113.7, 109.1, 106.7, 55.7 (OMe) ppm; HRMS (ESI^+^): *m/z* [M]^+^ calc. C_16_H_13_BrN_2_O_4_S_2_ 439.9495, found 440.9502 for [M + H]^+^.

*N-[4-(2-Hydroxy-4-methoxyphenyl)thiazol-2-yl]-4-nitrobenzenesulfonamide* (**5g**): Yield 84%. Orange solid. IR (film): ν 3607.7, 2685.6, 1554.65, 1360.65, 1325.25, 1267.54, 964.95 cm^−1^. ^1^H-NMR (400 MHz, CDCl_3_): δ 8.06 (d, *J* = 8.0 Hz, 2H, 2 × ArH), 7.59 (d, *J* = 8.0 Hz, 2H, 2 × ArH), 7.29 (d, *J* = 8.6 Hz, 1H, H-6), 6.90 (s, 1H, H-3), 6.77 (d, *J* = 8.6 Hz, 1H, H-5), 6.32 (s, 1H, SCH), 3.76 (s, 3H, OMe) ppm. ^13^C-NMR (100 MHz, CDCl_3_): δ 166.2, 159.9, 150.5, 146.6, 131.1, 129.7, 124.8, 114.0, 109.4, 106.7, 55.8 (OMe) ppm; HRMS (ESI^+^): *m/z* [M]^+^ calc. for C_16_H_13_N_3_O_6_S_2_ 407.0240, found 408.0247 for [M + H]^+^.

### 4.7. Cell Culture 

Human lung adenocarcinoma A549 (BCRC-60074) cell line was purchased from the Bioresource Collection and Research Center (BCRC, Hsinchu, Taiwan). The cell line was initially established through explant cultures of lung carcinomatous tissue from a 58-year-old Caucasian male. The A549 cells were cultured in 10% fetal bovine serum (HyClone, GE Healthcare Life Science, Logan, UT, USA) and 90% Ham's F12K medium (Gibco, Thermo Fisher Scientific, Waltham, MA, USA) containing L-glutamine (2.0 mM) and sodium bicarbonate (1.5 g/L). Cells were cultured in a T-75 flask with a feeding cycle of 2 days. The humidified atmosphere with 5% CO_2_ kept at 37 °C was provided in cell incubator. 

### 4.8. Cytotoxicity of APDs

A549 cells were seeded into 96-well tissue culture plates at a concentration of 5 × 10^3^ cells/well for overnight growth to evaluate the cytotoxicity of APDs. Subsequently, A549 cells were treated with serial concentrations of eleven APDs. After 24 h of incubation, cell viability was determined using WST-1 assays (Abcam, Cambridge, MA, USA). Briefly, 10 µL of WST-1 reagent was added to each well and incubated for 2 h at 37 °C prior to quantification. The absorbance at 450 nm was measured by a VersaMax™ ELISA Microplate Reader (Molecular Devices, Sunnyvale, CA, USA) using a reference absorbance at 600 nm for each well.

### 4.9. Preliminary Screening of APDs Against LPS-Activated A549 Cells

Preliminary screening procedure to evaluate a series of APDs against LPS-induced inflammation was established with activated A549 cells. Before challenge with LPS, A549 cells were seeded into 24-well plate (5 × 10^4^ cells/well) for overnight. Twenty-four hours after challenge with LPS (1.0 µg/mL), low and high doses of APDs, Dexa, and paeonol (1.0 and 10 μg/mL) were added to activated A549 cells. Forty-eight hours after challenge with LPS, the secreted MCP-1 and IL-6 in the conditioned media from this system was measured by a commercial ELISA kit (R&D Systems, Minneapolis, MN, USA) according to the manufacturer’s instructions.

### 4.10. Animal Model of Acute Lung Injury

Adult male Sprague-Dawley rats weighing 250–300 g were obtained from the BioLASCO Breeding Center (Yi-Lan, Taiwan). Animals were housed in isolated ventilation cabinets (kept at 22–26 °C with 30–70% humidity) with a 12 h light/dark cycle and free access to food and water. Animal experiments were performed according to principles stated in the “Guide for the Care and Use of Laboratory Animals” and were approved by the Institutional Animal Care and Use Committee (IACUC) of the Agricultural Technology Research Institute (approved protocol number: 104033). Animals were randomly assigned to ten groups. Each group comprised six rats, including a negative control group (non-challenged; PBS group), a positive control group (8 mg LPS /kg; LPS group), and Dexa (1.0 and 5.0 mg/kg; Dexa (1) and Dexa (5) groups), paeonol (25 and 50 mg/kg; P (25) and P (50) groups), compound **4** (25 and 50 mg/kg; P-4 (25) and P-4 (50) groups) and compound **4.HCl** (25 and 50 mg/kg; P-4.HCl (25) and P-4.HCl (50) groups) as treatment groups. Figure 4 shows experimental design and groups. Animals were anesthetized using inhaled 2% isoflurane (Halocarbon Laboratories, River Edge, NJ, USA) in 0.5 L/min of air before challenge. Intratracheal administration of LPS (0.5 mL/rat) was performed by inserting a MicroSprayer^®^ Aerosolizer (Model IA-1B, Penn-Century, Wyndmoor, PA, USA) into the trachea. The microsprayer was subsequently removed, and animals were then placed in the vertical position and gently rotated for 30 s to distribute the spray homogenously throughout the lungs. The rats were treated with testing chemicals (APDs, Dexa and paeonol) through intraperitoneal (i.p.) injection at one hour after the LPS challenge and sacrificed at 8 h after the LPS challenge. The other two control groups (PBS and LPS group) were administered with vehicle (DMSO) through i.p. injection. 

### 4.11. Total Cell Counts in BALF 

Rats were euthanized at 8 h after the LPS challenge, and bronchoalveolar lavage fluid (BALF) was collected as previously described [9,10]. Briefly, the right main bronchus was ligated, and a catheter was inserted into the trachea. Double-lavage procedures were performed using 0.5 mL of normal saline to pass through the catheter twice. The BALF samples were kept on ice prior to cell counting. Total cell counts in the BALF were determined with a cell counter (Coulter Inc., Miami, FL, USA). 

### 4.12. Total Proteins, IL-6 and MCP-1 in BALF

Quantification of total proteins in the BALF samples was performed by a BCA protein assay (Thermo Scientific, Rockford, IL, USA) with bovine serum albumin solution as calibration standard. For quantification of chemokines and cytokines in BALF, commercial ELISA kits for rat MCP-1 and IL-6 were purchased from Bioscience Systems (San Diego, CA, USA). 

### 4.13. Histopathological Examination

The right lung tissues from 3 lobes of each rat were collected randomly and fixed with 10% neutral buffered paraformaldehyde (PFA) for 18 h. Fixed tissues were embedded in paraffin and dissected into 3-µm thick sections. Hematoxylin and eosin (H&E) staining was used for histopathological examination. Protein exudation, infiltration of red blood cells, mononuclear, and neutrophilic cells were examined by a pathologist and a medical doctor in pulmonary medicine using an Eclipse Ni-U microscope (Nikon, Tokyo, Japan) at 100× magnification. 

### 4.14. Statistical Analysis

All data are presented as the mean ± standard deviation (SD). Figures and statistical analysis were plotted and performed by GraphPad Prism 5 (GraphPad Software Inc., La Jolla, CA, USA). The Mann-Whitney test was used for comparisons between two groups, including differences between particularly experimental group and positive control (LPS-activated) group. Statistical analysis was performed using non-parametric statistics by IBM SPSS Statistics (Version 22.0, Armonk, NY, USA). All comparisons were considered significantly different at *p* values less than 0.05.

## 5. Conclusions

To evaluate a series of APDs for treating ALI, eleven APDs were prepared in this work and evaluated using LPS-activated A549 cell model for preliminary screening integrated with ALI-bearing rat model for verification. The best compound, 2-(2-aminothiazol-4-yl)-5-methoxyphenol (**4**), can effectively alleviate ALI in rats through comprehensively inhibition of chemokines/cytokines, attenuating neutrophil infiltration and eliminating protein exudation in alveolar space. The achievement of this study gives us insight into developing plant-derived compounds to avoid human from suffering lethal ALI and ARDS.

## Figures and Tables

**Table 1:** Secretion of MCP-1 in conditioned media of LPS-activated A549 cells treated with low (1.0 µg/mL) and high (10 µg/mL) concentrations of Dexa, paeonol, and APDs.

Drugs (Symbols)	Secretion of MCP-1 in Conditioned Media (pg/mL)	
	Low Dose Treatment	High Dose Treatment
Dexamethasone (Dexa)	2669 ± 578 *	2538 ± 343 *
Paeonol (P)	3938 ± 106	3584 ± 329
2 (P-2)	4332 ± 332	3610 ± 505
3 (P-3)	3618 ± 267	4446 ± 201
4 (P-4)	2724 ± 254 *	2077 ± 111 *
4.HCl (P-4.HCl)	3323 ± 261	2826 ± 362 *
5a (P-5a)	3980 ± 424	3225 ± 76
5b (P-5b)	3983 ± 120	3786 ± 131
5c (P-5c)	4238 ± 464	3793 ± 176
5d (P-5d)	4140 ± 416	4118 ± 372
5e (P-5e)	4002 ± 125	3893 ± 312
5f (P-5f)	4050 ± 2.3	4334 ± 544
5g (P-5g)	4354 ± 155	4628 ± 89

Significant differences of MCP-1 secretion between positive control group and each treatment group at *p* values less than 0.05 were annotated with *. The MCP-1 concentration in positive control (LPS-activated) groups was 4163.5 pg/mL.

## Supplementary Materials

The following are available online, Figure S1-S20: The ^1^H- and ^13^C-NMR Spectra of compounds **2, 3, 4, 5a, 5b, 5c, 5d, 5e, 5f** and **5g**.

## References

[1] Ghosh N., Ali A., Ghosh R., Das S., Mandal S.C., Pal M. (2016). Chronic Inflammatory Diseases: Progress and Prospect with Herbal Medicine. Curr. Pharm. Des..

[2] Feng Q., Ren Y., Wang Y., Ma H., Xu J., Zhou C., Yin Z., Luo L. (2008). Anti-inflammatory effect of SQC-beta-CD on lipopolysaccharide-induced acute lung injury. J. Ethnopharmacol..

[3] Ma C., Zhu L., Wang J., He H., Chang X., Gao J., Shumin W., Yan T. (2015). Anti-inflammatory effects of water extract of Taraxacum mongolicum hand.-Mazz on lipopolysaccharide-induced inflammation in acute lung injury by suppressing PI3K/Akt/mTOR signaling pathway. J. Ethnopharmacol..

[4] Pan L., Dai M. (2009). Paeonol from Paeonia suffruticosa prevents TNF-alpha-induced monocytic cell adhesion to rat aortic endothelial cells by suppression of VCAM-1 expression. Phytomedicine.

[5] Nizamutdinova I.T., Oh H.M., Min Y.N., Park S.H., Lee M.J., Kim J.S., Yean M.H., Kang S.S., Kim Y.S., Chang K.C. (2007). Paeonol suppresses intercellular adhesion molecule-1 expression in tumor necrosis factor-alpha-stimulated human umbilical vein endothelial cells by blocking p38, ERK and nuclear factor-kappaB signaling pathways. Int. Immunopharmacol..

[6] Chae H.S., Kang O.H., Lee Y.S., Choi J.G., Oh Y.C., Jang H.J., Kim M.S., Kim J.H., Jeong S.I., Kwon D.Y. (2009). Inhibition of LPS-induced iNOS, COX-2 and inflammatory mediator expression by paeonol through the MAPKs inactivation in RAW 264.7 cells. Am. J. Chin. Med..

[7] Liu M.H., Lin A.H., Lee H.F., Ko H.K., Lee T.S., Kou Y.R. (2014). Paeonol attenuates cigarette smoke-induced lung inflammation by inhibiting ROS-sensitive inflammatory signaling. Mediators Inflamm..

[8] Jamal J., Mustafa M.R., Wong P.F. (2014). Paeonol protects against premature senescence in endothelial cells by modulating Sirtuin 1 pathway. J. Ethnopharmacol..

[9] Fu P.K., Wu C.L., Tsai T.H., Hsieh C.L. (2012). Anti-inflammatory and anticoagulative effects of paeonol on LPS-induced acute lung injury in rats. Evid. Based Complement. Alternat. Med..

[10] Fu P.K., Yang C.Y., Tsai T.H., Hsieh C.L. (2012). Moutan cortex radicis improves lipopolysaccharide-induced acute lung injury in rats through anti-inflammation. Phytomedicine.

[11] Qin D.D., Yang Z.Y., Zhang F.H., Du B., Wang P., Li T.R. (2010). Evaluation of the antioxidant, DNA interaction and tumor cell cytotoxicity activities of Copper(II) complexes with Paeonol Schiff-base. Inorg. Chem. Commun..

[12] Huang T.J., Chuang H., Liang Y.C., Lin H.H., Horng J.C., Kuo Y.C., Chen C.W., Tsai F.Y., Yen S.C., Chou S.C. (2015). Design, synthesis, and bioevaluation of paeonol derivatives as potential anti-HBV agents. Eur. J. Med. Chem..

[13] Pao K.C., Zhao J.F., Lee T.S., Huang Y.P., Han C.C., Huang L.C.S., Wu K.H., Hsu M.H. (2015). Low-dose paeonol derivatives alleviate lipid accumulation. RSC Adv..

[14] Tsai C.Y., Kapoor M., Huang Y.P., Lin H.H., Liang Y.C., Lin Y.L., Huang S.C., Liao W.N., Chen J.K., Huang J.S. (2016). Synthesis and Evaluation of Aminothiazole-Paeonol Derivatives as Potential Anticancer Agents. Molecules.

[15] Van Vliet L.A., Rodenhuis N., Wikstrom H., Pugsley T.A., Serpa K.A., Meltzer L.T., Heffner T.G., Wise L.D., Lajiness M.E., Huff R.M. (2000). Thiazoloindans and thiazolobenzopyrans: a novel class of orally active central dopamine (partial) agonists. J. Med. Chem..

[16] Lee Y.S., Chuang S.H., Huang L.Y., Lai C.L., Lin Y.H., Yang J.Y., Liu C.W., Yang S.C., Lin H.S., Chang C.C. (2014). Discovery of 4-aryl-N-arylcarbonyl-2-aminothiazoles as Hec1/Nek2 inhibitors. Part I: optimization of in vitro potencies and pharmacokinetic properties. J. Med. Chem..

[17] Das D., Sikdar P., Bairagi M. (2016). Recent developments of 2-aminothiazoles in medicinal chemistry. Eur. J. Med. Chem..

[18] Ware L.B., Matthay M.A. (2000). The acute respiratory distress syndrome. N. Engl. J. Med..

[19] Ranieri V.M., Rubenfeld G.D., Thompson B.T., Ferguson N.D., Caldwell E., Fan E., Camporota L., Slutsky A.S. (2012). Acute respiratory distress syndrome: the Berlin Definition. JAMA.

[20] Fujishima S., Gando S., Daizoh S., Kushimoto S., Ogura H., Mayumi T., Takuma K., Kotani J., Yamashita N., Tsuruta R. (2016). Infection site is predictive of outcome in acute lung injury associated with severe sepsis and septic shock. Respirology.

[21] Matthay M.A., Brower R.G., Carson S., Douglas I.S., Eisner M., Hite D., Holets S., Kallet R.H., Liu K.D., MacIntyre N. (2011). Randomized, placebo-controlled clinical trial of an aerosolized beta(2)-agonist for treatment of acute lung injury. Am. J. Respir. Crit. Care Med..

[22] Xiong B., Wang C., Tan J., Cao Y., Zou Y., Yao Y., Qian J., Rong S., Huang Y., Huang J. (2016). Statins for the prevention and treatment of acute lung injury and acute respiratory distress syndrome: A systematic review and meta-analysis. Respirology.

[23] Kor D.J., Carter R.E., Park P.K., Festic E., Banner-Goodspeed V.M., Hinds R., Talmor D., Gajic O., Ware L.B., Gong M.N. (2016). Effect of Aspirin on Development of ARDS in At-Risk Patients Presenting to the Emergency Department: The LIPS-A Randomized Clinical Trial. JAMA.

[24] Matute-Bello G., Frevert C.W., Martin T.R. (2008). Animal models of acute lung injury. Am. J. Physiol. Lung Cell Mol. Physiol..

[25] Rocco P.R., Nieman G.F. (2016). ARDS: what experimental models have taught us. Intensive Care Med..

[26] Chu C., Ren H., Xu N., Xia L., Chen D., Zhang J. (2016). Eupatorium lindleyanum DC. sesquiterpenes fraction attenuates lipopolysaccharide-induced acute lung injury in mice. J. Ethnopharmacol..

[27] Dreyfuss D., Ricard J.D. (2005). Acute lung injury and bacterial infection. Clin. Chest Med..

[28] Sapru A., Wiemels J.L., Witte J.S., Ware L.B., Matthay M.A. (2006). Acute lung injury and the coagulation pathway: Potential role of gene polymorphisms in the protein C and fibrinolytic pathways. Intensive Care Med..

[29] Fujishima S. (2014). Pathophysiology and biomarkers of acute respiratory distress syndrome. J. Intensive Care Med..

[30] Lien D.C., Wagner Jr W.W., Capen R.L., Haslett C., Hanson W.L., Hofmeister S.E., Henson P.M., Worthen G.S. (1987). Physiological neutrophil sequestration in the lung: visual evidence for localization in capillaries. J. Appl. Physiol..

[31] Worthen G.S., Schwab B., Elson E.L., Downey G.P. (1989). Mechanics of stimulated neutrophils: cell stiffening induces retention in capillaries. Science.

[32] Meduri G.U., Kohler G., Headley S., Tolley E., Stentz F., Postlethwaite A. (1995). Inflammatory cytokines in the BAL of patients with ARDS. Persistent elevation over time predicts poor outcome. Chest.

[33] Bhargava M., Wendt C.H. (2012). Biomarkers in acute lung injury. Translational Research.

[34] Warren J.S., Jones M.L., Flory C.M. (1993). Analysis of monocyte chemoattractant protein 1-mediated lung injury using rat lung organ cultures. Am. J. Pathol..

[35] Han Z., Boyle D.L., Chang L., Bennett B., Karin M., Yang L., Manning A.M., Firestein G.S. (2001). c-Jun N-terminal kinase is required for metalloproteinase expression and joint destruction in inflammatory arthritis. J. Clin. Investig..

[36] Moore T.A., Standiford T.J. (2001). Cytokine immunotherapy during bacterial pneumonia: from benchtop to bedside. Semin. Respir. Infect..

[37] Yong K.K., Chang J.H., Chien M.H., Tsao S.M., Yu M.C., Bai K.J., Tsao T.C., Yang S.F. (2016). Plasma monocyte chemoattractant protein-1 level as a predictor of the severity of community-acquired pneumonia. Int. J. Mol. Sci..

[38] Chen J.K., Ho C.C., Chang H., Lin J.F., Yang C.S., Tsai M.H., Tsai H.T., Lin P. (2015). Particulate nature of inhaled zinc oxide nanoparticles determines systemic effects and mechanisms of pulmonary inflammation in mice. Nanotoxicology.

[39] Dos Santos G., Rogel M.R., Baker M.A., Troken J.R., Urich D., Morales-Nebreda L., Sennello J.A., Kutuzov M.A., Sitikov A., Davis J.M. (2015). Vimentin regulates activation of the NLRP3 inflammasome. Nat. Commun..

[40] Liang J., Zhang Y., Xie T., Liu N., Chen H., Geng Y., Kurkciyan A., Mena J.M., Stripp B.R., Jiang D. (2016). Hyaluronan and TLR4 promote surfactant-protein-C-positive alveolar progenitor cell renewal and prevent severe pulmonary fibrosis in mice. Nat. Med..

[41] O'Grady N.P., Preas H.L., Pugin J., Fiuza C., Tropea M., Reda D., Banks S.M., Suffredini A.F. (2001). Local inflammatory responses following bronchial endotoxin instillation in humans. Am. J. Respir. Crit. Care Med..

[42] Wu C.L., Lin L.Y., Yang J.S., Chan M.C., Hsueh C.M. (2009). Attenuation of lipopolysaccharide-induced acute lung injury by treatment with IL-10. Respirology.

[43] Fu P.K. (2012). Anti-inflammatory and anticoagulative effects of Moutan Cortex Radices and paeonol on LPS-induced acute lung injury in rats. Ph.D. Thesis.

[44] Tatsumi S., Mabuchi T., Abe T., Xu L., Minami T., Ito S. (2004). Analgesic effect of extracts of Chinese medicinal herbs Moutan cortex and Coicis semen on neuropathic pain in mice. Neurosci. Lett..

[45] Yoshikawa M., Ohta T., Kawaguchi A., Matsuda H. (2000). Bioactive constituents of Chinese natural medicines. V. Radical scavenging effect of Moutan Cortex. (1): Absolute stereostructures of two monoterpenes, paeonisuffrone and paeonisuffral. Chem. Pharm. Bull..

[46] Zhu T.H., Cao S.W., Yu Y.Y. (2013). Synthesis, characterization and biological evaluation of paeonol thiosemicarbazone analogues as mushroom tyrosinase inhibitors. Int. J. Biol. Macromol..

[47] Ortiz-Diaz E., Festic E., Gajic O., Levitt J.E. (2013). Emerging pharmacological therapies for prevention and early treatment of acute lung injury. Semin. Respir. Crit. Care Med..

[48] Bosma K.J., Taneja R., Lewis J.F. (2010). Pharmacotherapy for prevention and treatment of acute respiratory distress syndrome: current and experimental approaches. Drugs.

[49] Johnson E.R., Matthay M.A. (2010). Acute lung injury: epidemiology, pathogenesis, and treatment. J. Aerosol Med. Pulm. Drug Deliv..

[50] Mayer-Scholl A., Averhoff P., Zychlinsky A. (2004). How do neutrophils and pathogens interact?. Curr. Opin. Microbiol..

[51] Olson T.S., Ley K. (2002). Chemokines and chemokine receptors in leukocyte trafficking. Am. J. Physiol. Regul. Integr. Comp. Physiol..

[52] Hampton M.B., Kettle A.J., Winterbourn C.C. (1998). Inside the neutrophil phagosome: oxidants, myeloperoxidase, and bacterial killing. Blood.

[53] Strieter R.M., Kunkel S.L. (1994). Acute lung injury: the role of cytokines in the elicitation of neutrophils. J. Investig. Med..

[54] Kobayashi A., Hashimoto S., Kooguchi K., Kitamura Y., Onodera H., Urata Y., Ashihara T. (1998). Expression of inducible nitric oxide synthase and inflammatory cytokines in alveolar macrophages of ARDS following sepsis. Chest.

[55] Shinbori T., Walczak H., Krammer P.H. (2004). Activated T killer cells induce apoptosis in lung epithelial cells and the release of pro-inflammatory cytokine TNF-alpha. Eur. J. Immunol..

[56] Balamayooran G., Batra S., Balamayooran T., Cai S., Jeyaseelan S. (2011). Monocyte chemoattractant protein 1 regulates pulmonary host defense via neutrophil recruitment during Escherichia coli infection. Infect. Immun..

