# Supplementary material for: Evaluation of LPS-Induced Acute Lung Injury Attenuation in Rats by Aminothiazole-Paeonol Derivatives
Source: Molecules. 2017 Sep 25;22(10):1605. doi: 10.3390/molecules22101605 (PMC6151495; doi:10.3390/molecules22101605)
Supplement: Supplementary file 1 [file molecules-22-01605-s001.pdf]

Supplementary Materials

Evaluation of Aminothiazole-Paeonol Derivatives to Attenuate LPS-induced Acute Lung Injury in Rats

Pin-Kuei Fu[1,2] , Chi-Yu Yang[3] , Su-Chin Huang[4] , Yu-Wen Hung[3] , Kee-Ching Jeng[5] , Ying-Pei Huang[6,7] , Hong Chuang[6,7] , Nai-Chun Huang[4] , Jei-Ping Li[4] , Ming-Hua Hsu[7,8] and Jen-Kun Chen[4,9,10*]

1 Department of Critical Care Medicine, Taichung Veterans General Hospital, Taichung, 40705, Taiwan; yetquen@gmail.com (P.-K. F.)

2 Department of Biotechnology, Hungkuang University, Taichung, 43302, Taiwan

3 Animal Technology Laboratory, Agriculture Technology Research Institute, Miaoli 35053, Taiwan; chiyu@mail.atri.org.tw (C.-Y. C.); pp33887788@gmail.com (Y.-W. H.)

4 Institute of Biomedical Engineering and Nanomedicine, National Health Research Institutes, Miaoli 35053, Taiwan; jkchen@nhri.org.tw (J.-K. C.); chin@nhri.org.tw (S.-C. H.); anai@nhri.org.tw (N.-C. H.); piny72@nhri.org.tw (J.-P. L.)

5 Department of Medical Research, Tungs' Taichung MetroHarbor Hospital, Taichung 43503, Taiwan; kcjeng@gmail.com (K.-C. J.)

6 Department of Chemistry, National Tsing Hua University, Hsinchu 30013, Taiwan; suzann800217@hotmail.com (Y.-P. H.); hom770706@gmail.com (H. C.)

7 Nuclear Science & Technology Development Center, National Tsing Hua University, Hsinchu 30013, Taiwan; mhhsu@mx.nthu.edu.tw (M.-H. H.)

8 Department of Chemistry, National Changhua University of Education, Changhua County 50007, Taiwan; minghuahsu@cc.ncue.edu.tw (M.-H. H.)

9 Graduate Institute of Life Sciences, National Defense Medical Center, Taipei 11490, Taiwan

10 School of Dentistry, National Defense Medical Center, Taipei 11490, Taiwan

* Correspondence: jkchen@nhri.org.tw ; Tel.: +886-3-724-6166 (ext. 38117); Fax: +886-3-758-6440

List of Contents

Fig. S1 The ^{1}H NMR Spectrum of Compound **2** in $CDCl_3$ (500 MHz)
Fig. S2 The ^{13}C NMR Spectrum of Compound **2** in $CDCl_3$ (125MHz)
Fig. S3 The ^{1}H NMR Spectrum of Compound **3** in DMSO (500 MHz)
Fig. S4 The ^{13}C NMR Spectrum of Compound **3** in DMSO (125MHz)
Fig. S5 The ^{1}H NMR Spectrum of Compound **4** in $CDCl_3$ (400 MHz)
Fig. S6 The ^{13}C NMR Spectrum of Compound **4** in $CDCl_3$ (100MHz)
Fig. S7 The ^{1}H NMR Spectrum of Compound **5a** in $CDCl_3$ (400 MHz)
Fig. S8 The ^{13}C NMR Spectrum of Compound **5a** in $CDCl_3$ (100MHz)
Fig. S9 The ^{1}H NMR Spectrum of Compound **5b** in $CDCl_3$ (400 MHz)
Fig. S10 The ^{13}C NMR Spectrum of Compound **5b** in $CDCl_3$ (100MHz)
Fig. S11 The ^{1}H NMR Spectrum of Compound **5c** in $CDCl_3$ (400 MHz)
Fig. S12 The ^{13}C NMR Spectrum of Compound **5c** in $CDCl_3$ (100MHz)
Fig. S13 The ^{1}H NMR Spectrum of Compound **5d** in $CDCl_3$ (400 MHz)
Fig. S14 The ^{13}C NMR Spectrum of Compound **5d** in $CDCl_3$ (100MHz)
Fig. S15 The ^{1}H NMR Spectrum of Compound **5e** in $CDCl_3$ (400 MHz)
Fig. S16 The ^{13}C NMR Spectrum of Compound **5e** in $CDCl_3$ (100MHz)
Fig. S17 The ^{1}H NMR Spectrum of Compound **5f** in $CDCl_3$ (400 MHz)
Fig. S18 The ^{13}C NMR Spectrum of Compound **5f** in $CDCl_3$ (100MHz)
Fig. S19 The ^{1}H NMR Spectrum of Compound **5g** in $CDCl_3$ (400 MHz)
Fig. S20 The ^{13}C NMR Spectrum of Compound **5g** in $CDCl_3$ (100MHz)

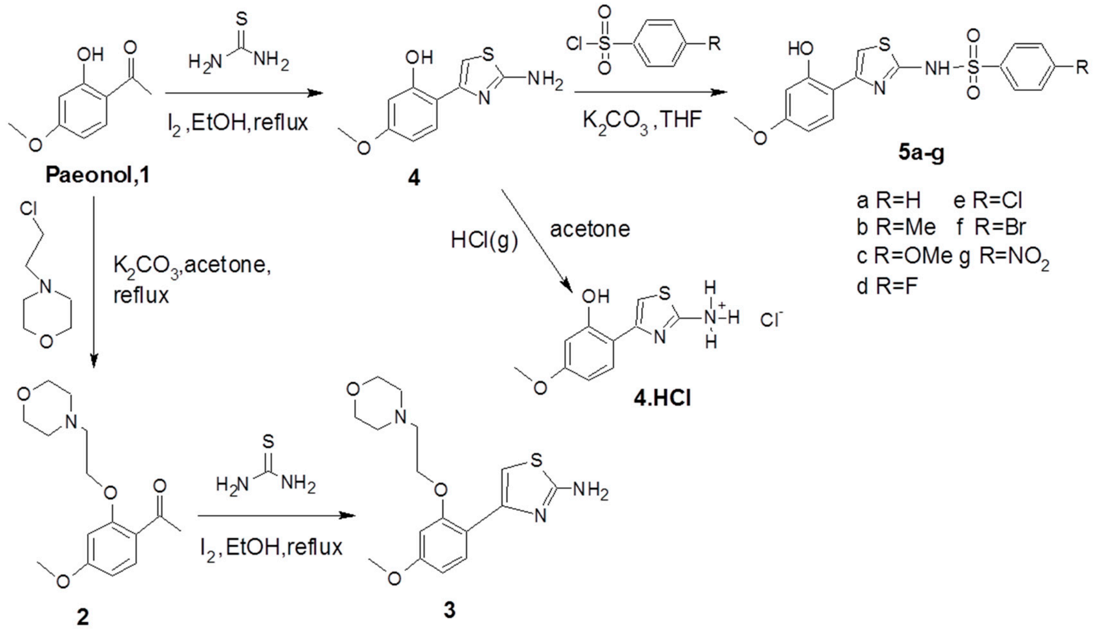

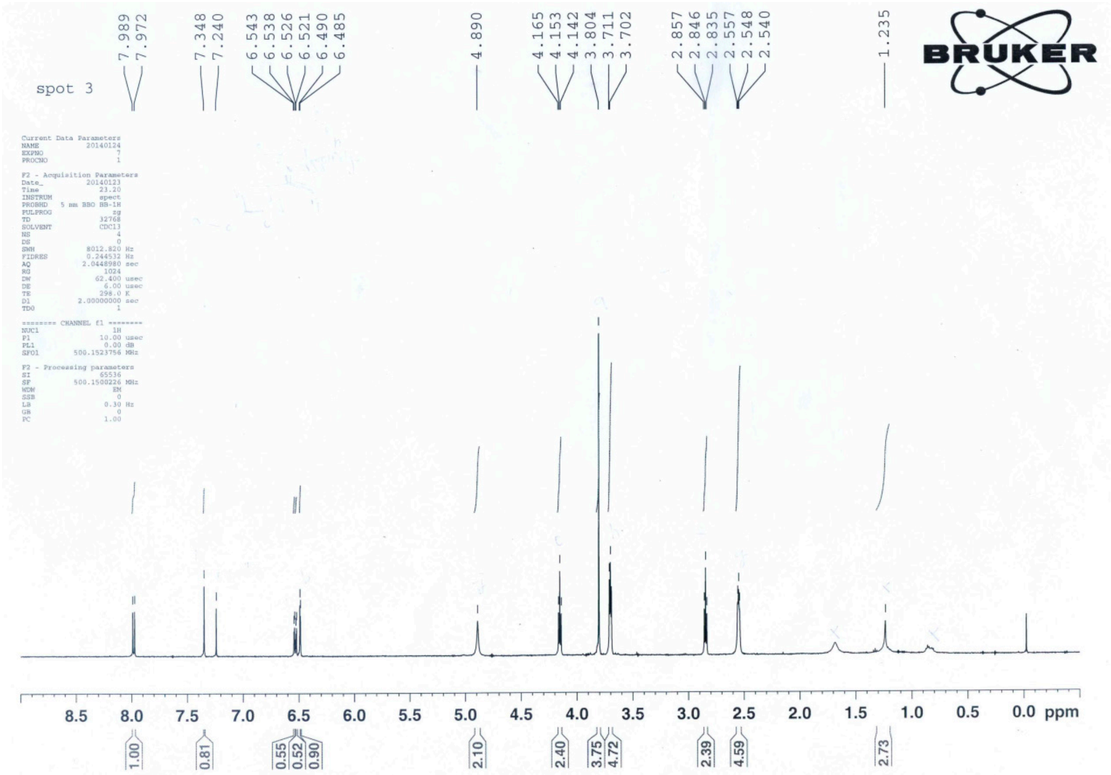

Fig. S1 The ^{1}H NMR Spectrum of Compound **2** in $CDCl_3$ (500 MHz)

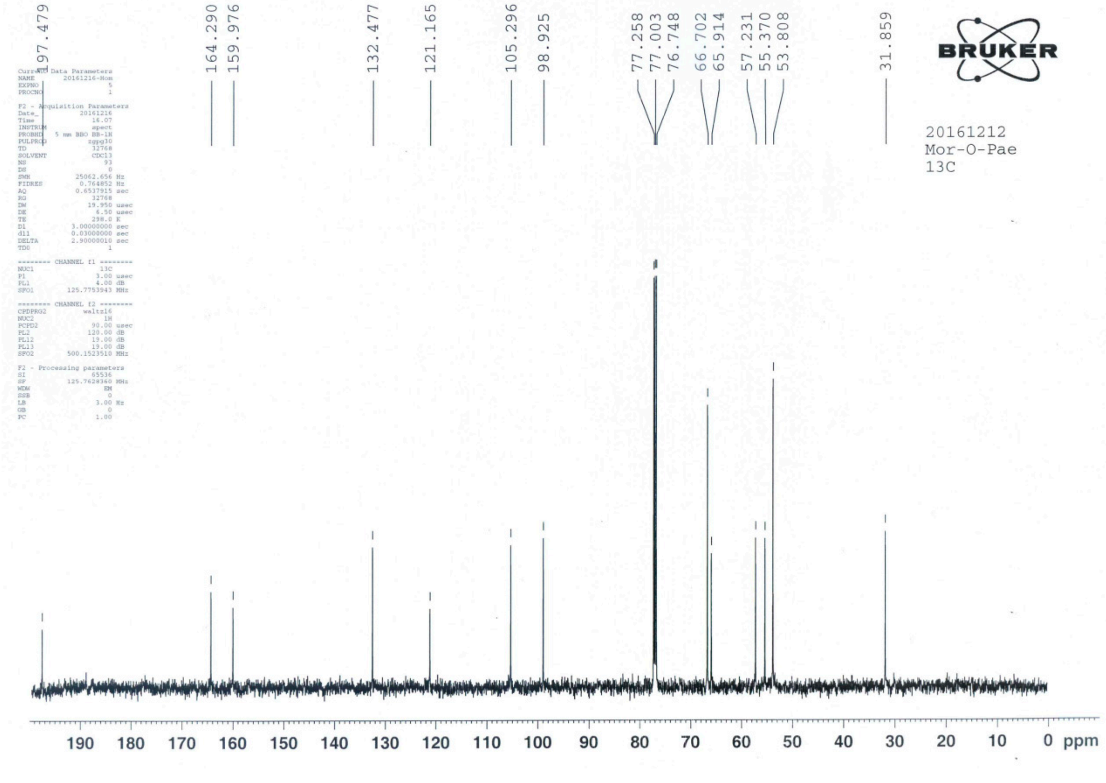

Fig. S2 The ^{13}C NMR Spectrum of Compound **2** in $CDCl_3$ (150MHz)

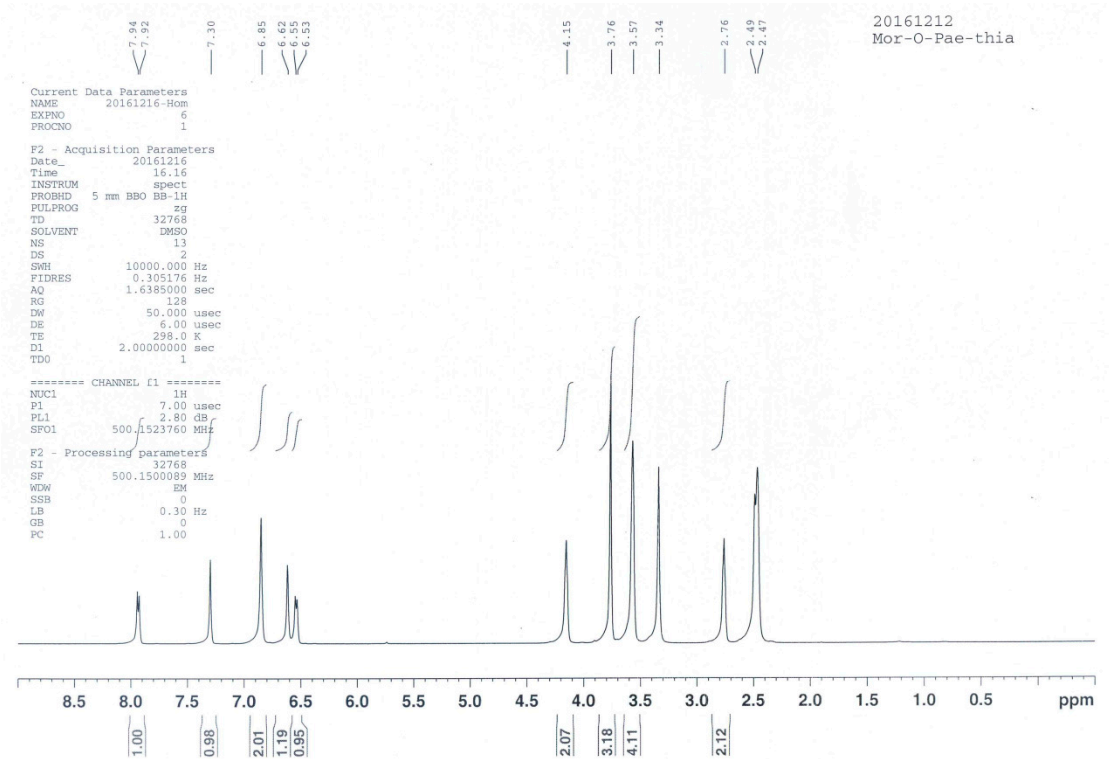

Fig. S3 The ^{1}H NMR Spectrum of Compound **3** in DMSO (500 MHz)

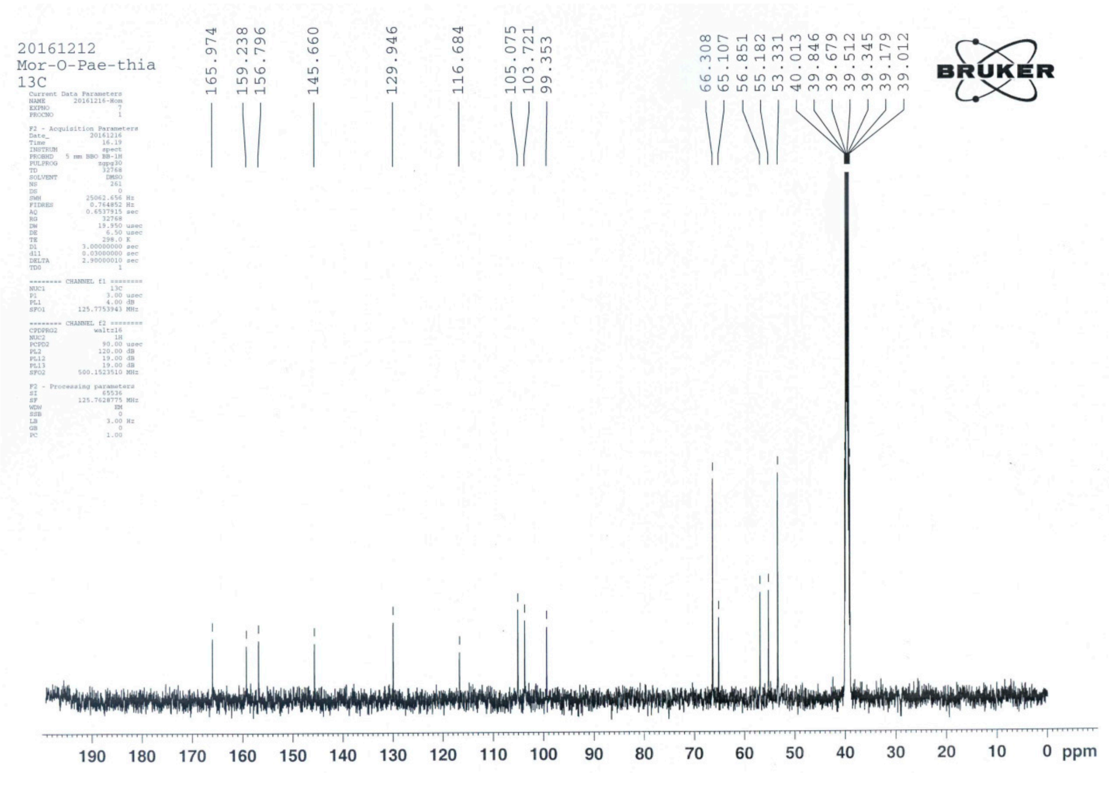

Fig. S4 The ^{13}C NMR Spectrum of Compound **3** in DMSO (125MHz)

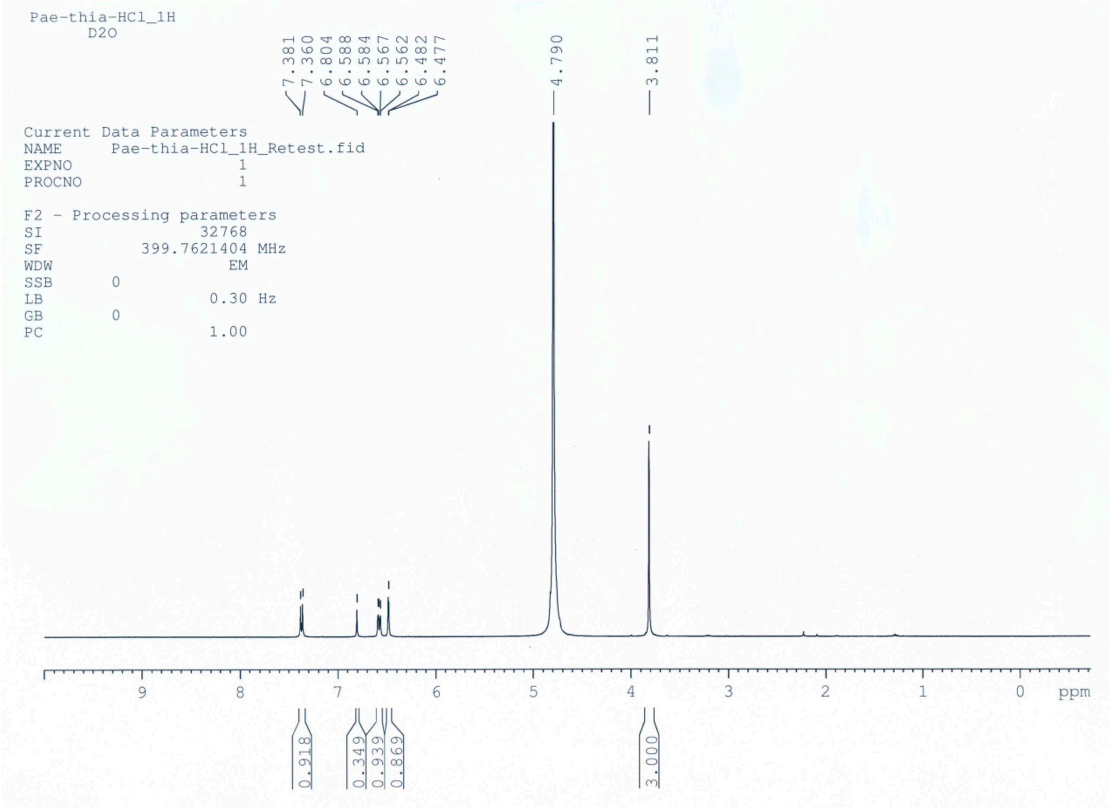

Fig. S5 The ^{1}H NMR Spectrum of Compound **4** in $CDCl_3$ (400 MHz)

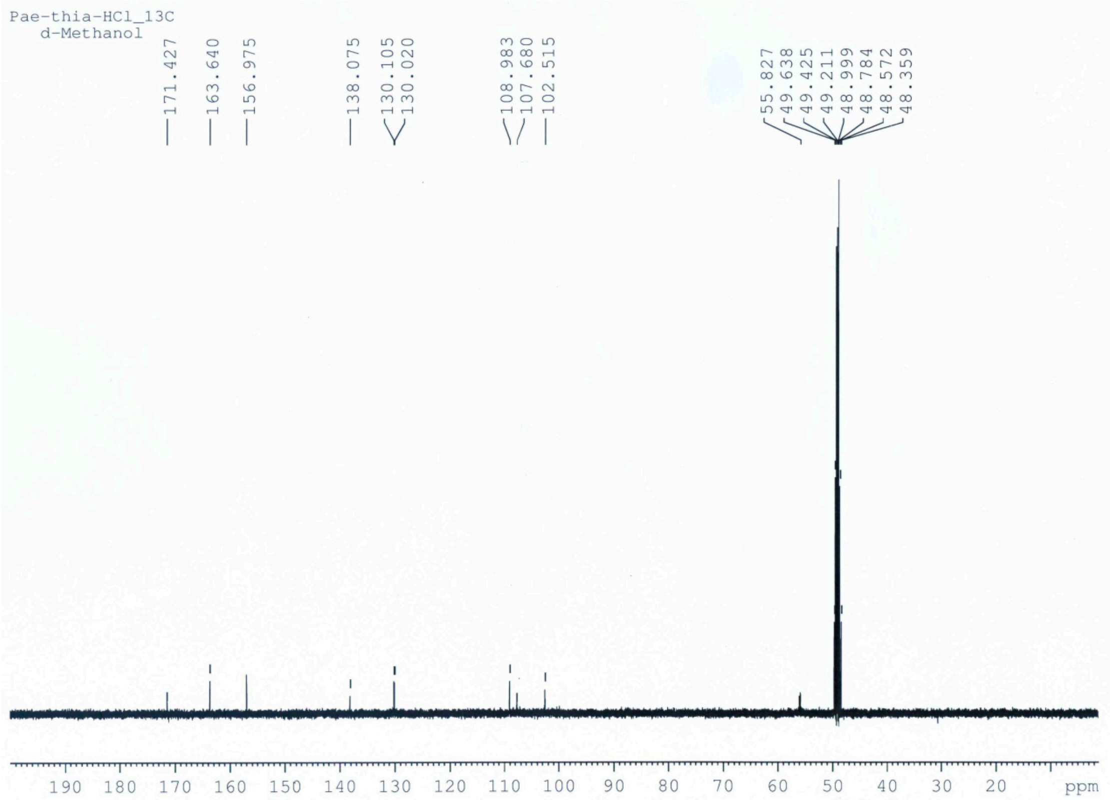

Fig. S6 The ^{13}C NMR Spectrum of Compound **4** in $CDCl_3$ (100MHz)

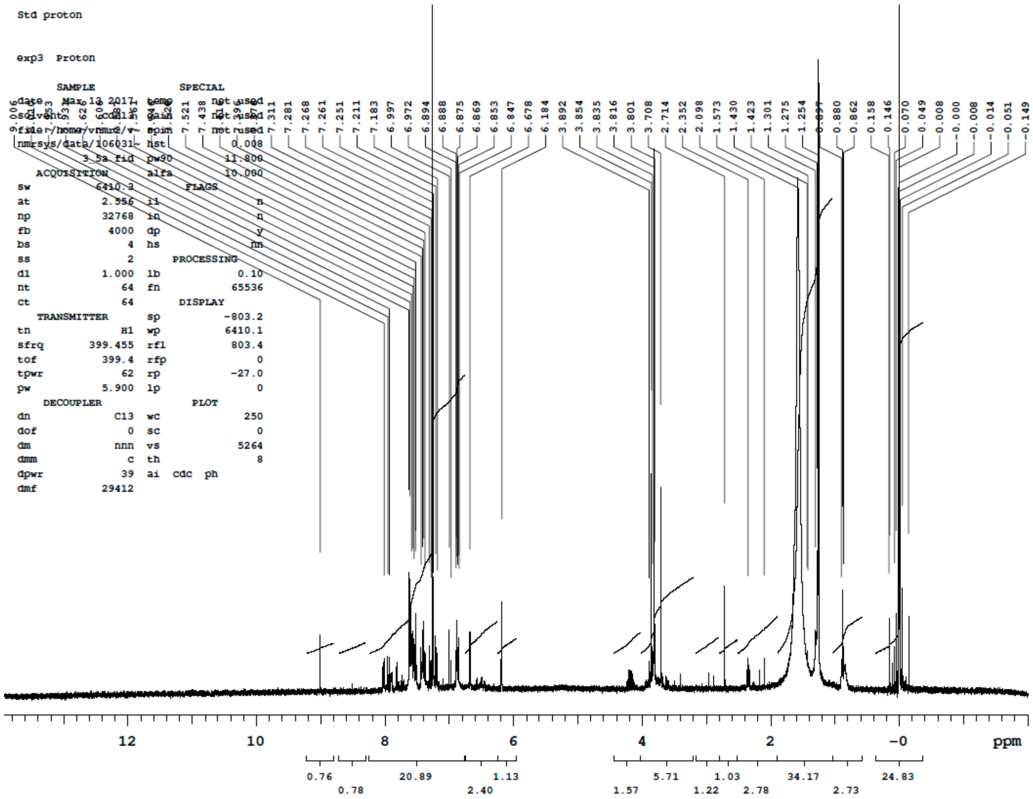

Fig. S7 The ^{1}H NMR Spectrum of Compound **5a** in $CDCl_3$ (400 MHz)

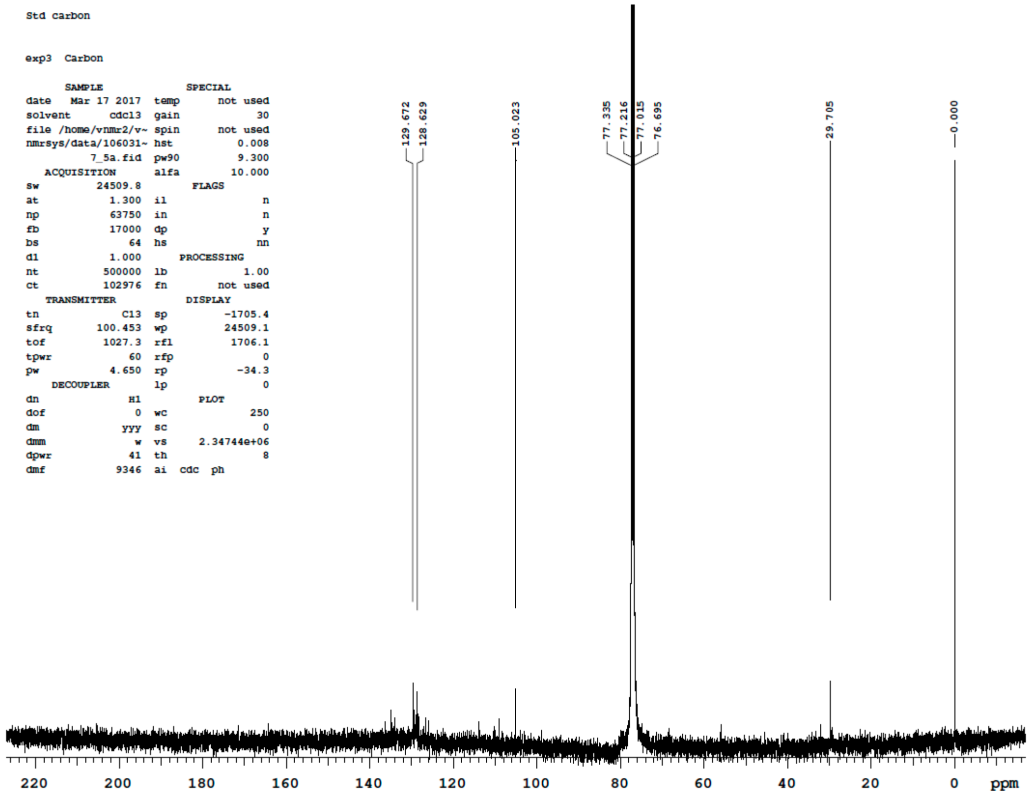

Fig. S8 The ^{13}C NMR Spectrum of Compound **5a** in $CDCl_3$ (100MHz)

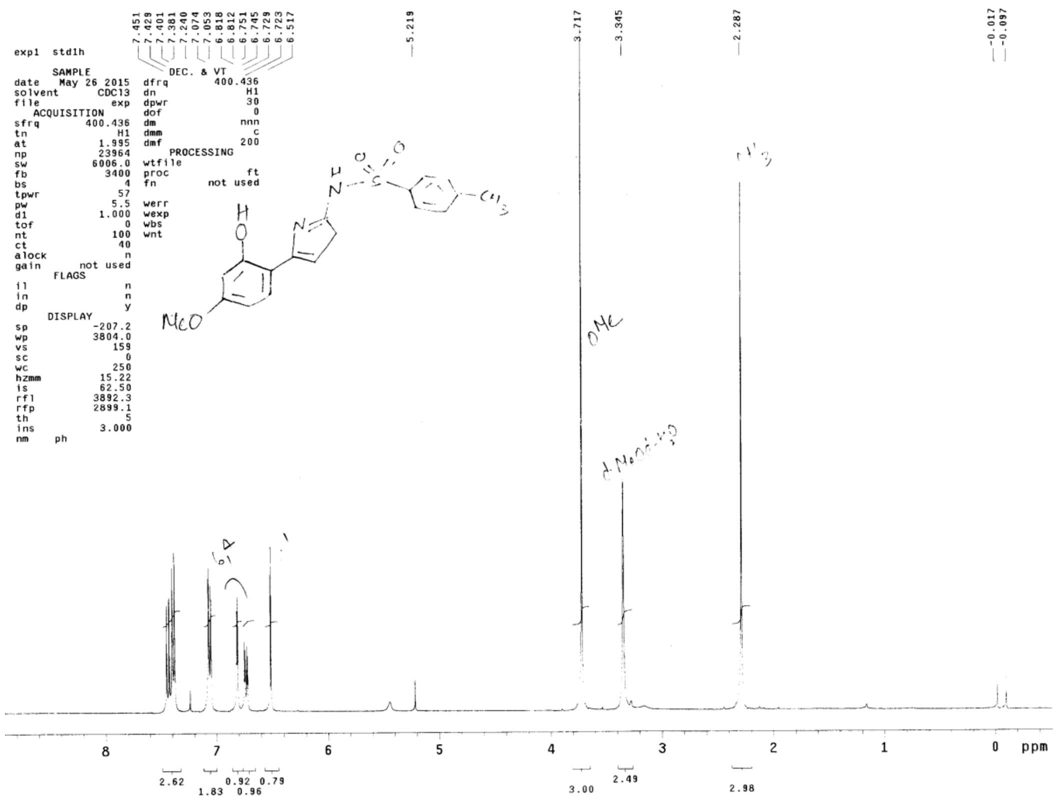

Fig. S9 The ^{1}H NMR Spectrum of Compound **5b** in $CDCl_3$ (400 MHz)

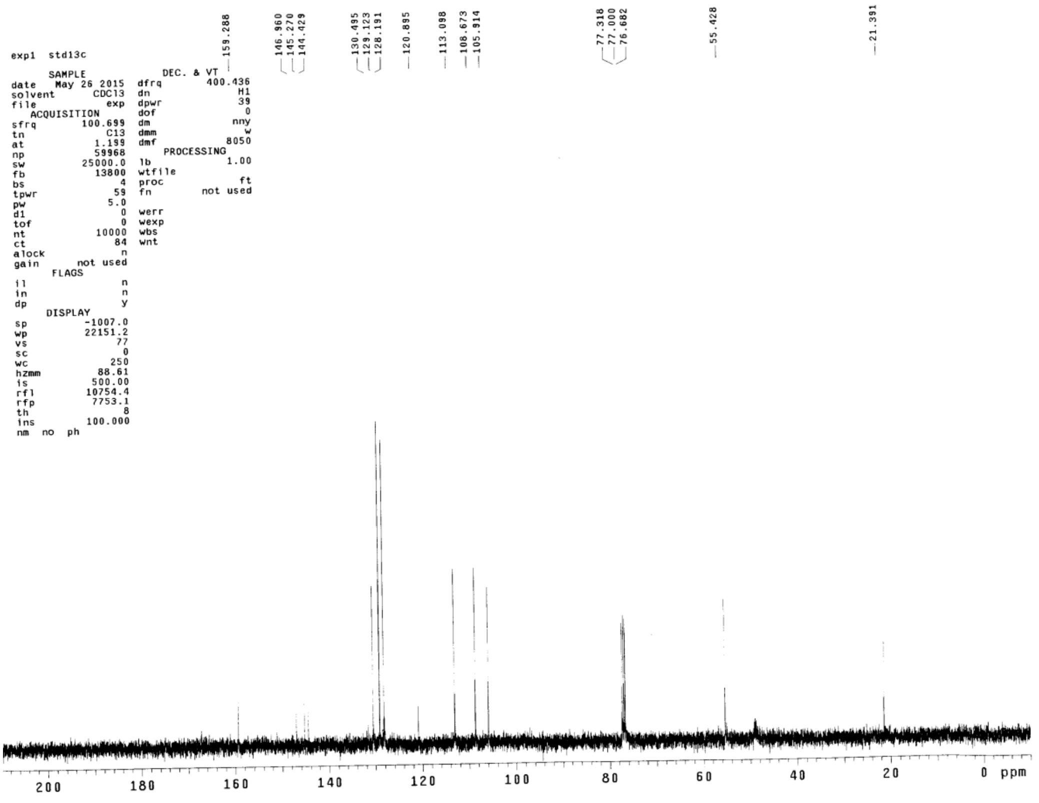

Fig. S10 The ^{13}C NMR Spectrum of Compound **5b** in $CDCl_3$ (100MHz)

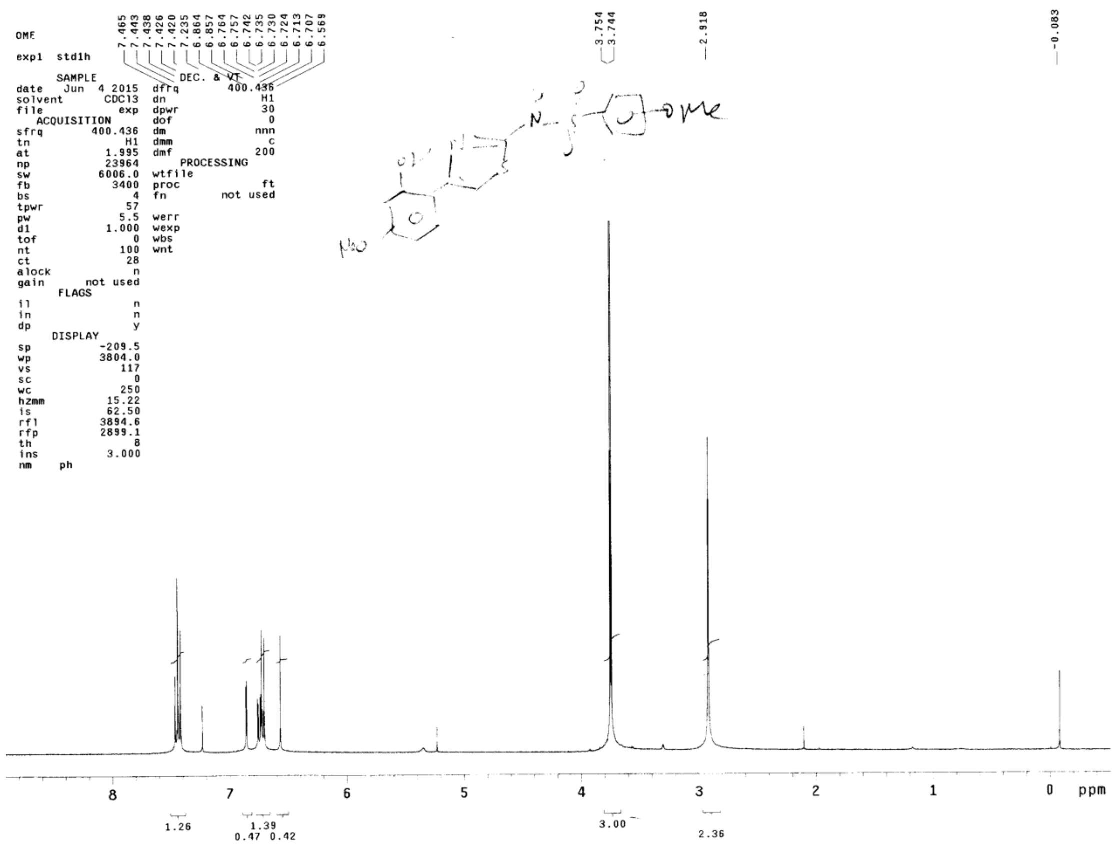

Fig. S11 The ^{1}H NMR Spectrum of Compound **5c** in $CDCl_3$ (400 MHz)

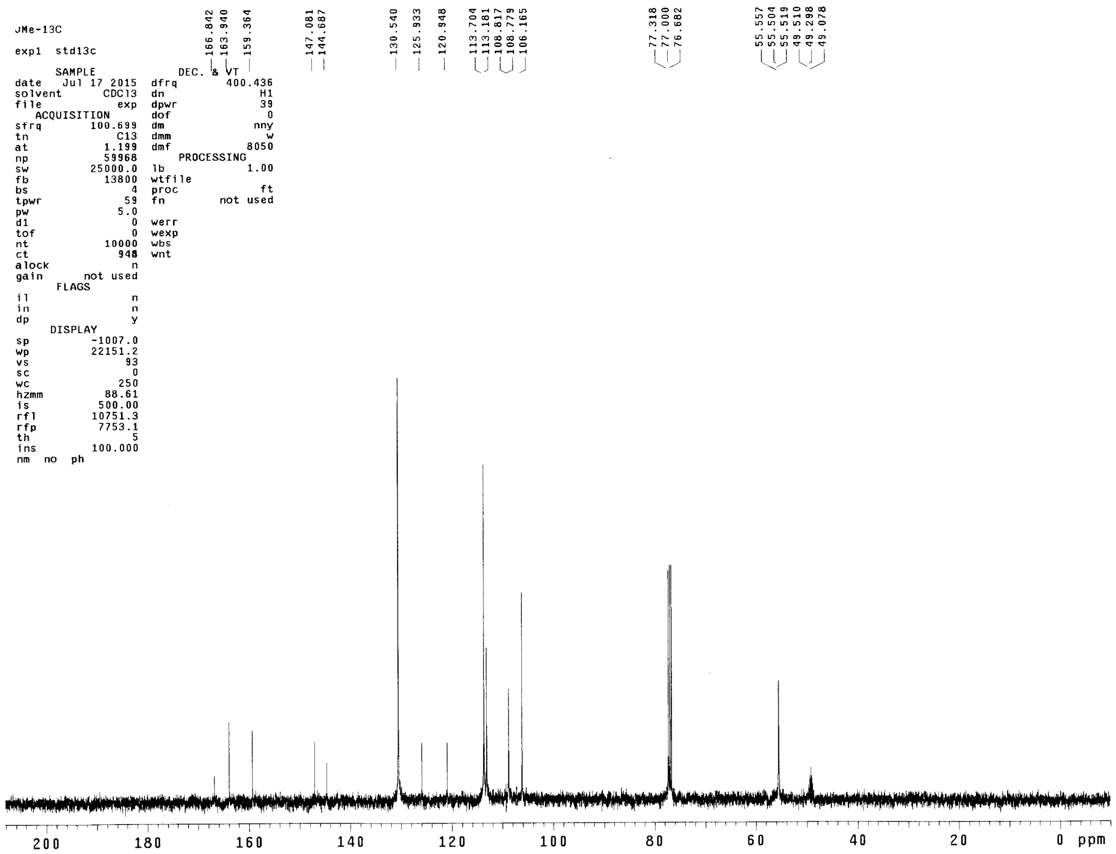

Fig. S12 The ^{13}C NMR Spectrum of Compound **5c** in $CDCl_3$ (100MHz)

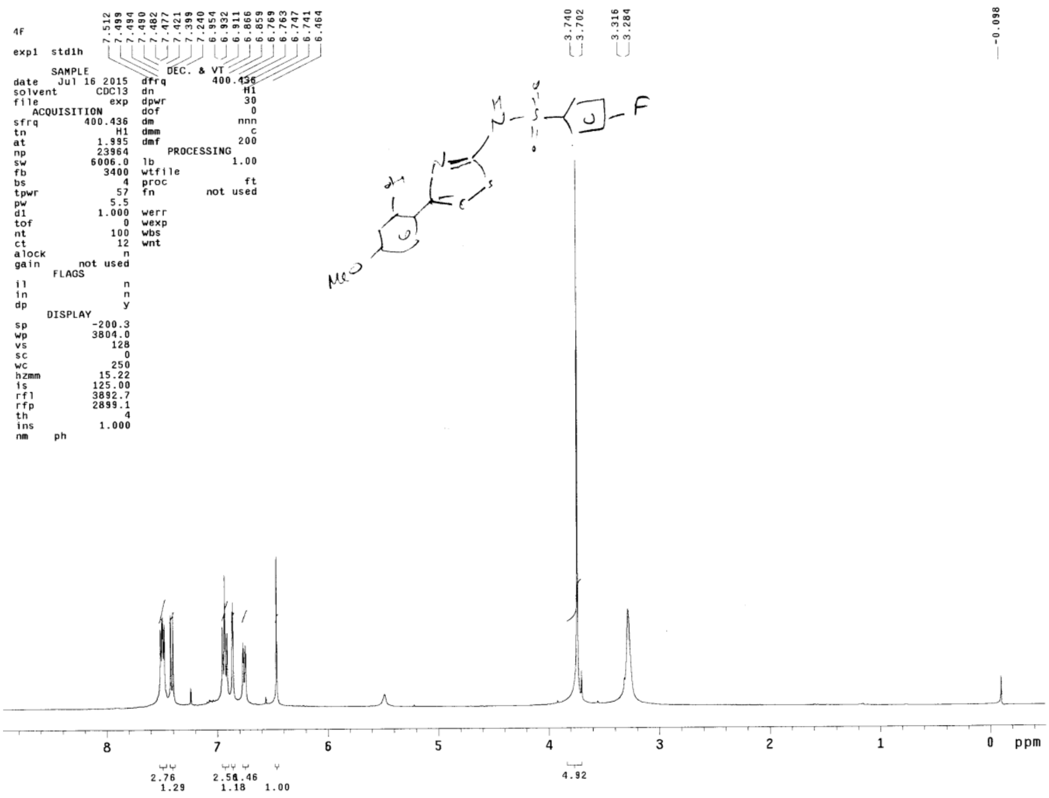

Fig. S13 The ^{1}H NMR Spectrum of Compound **5d** in $CDCl_3$ (400 MHz)

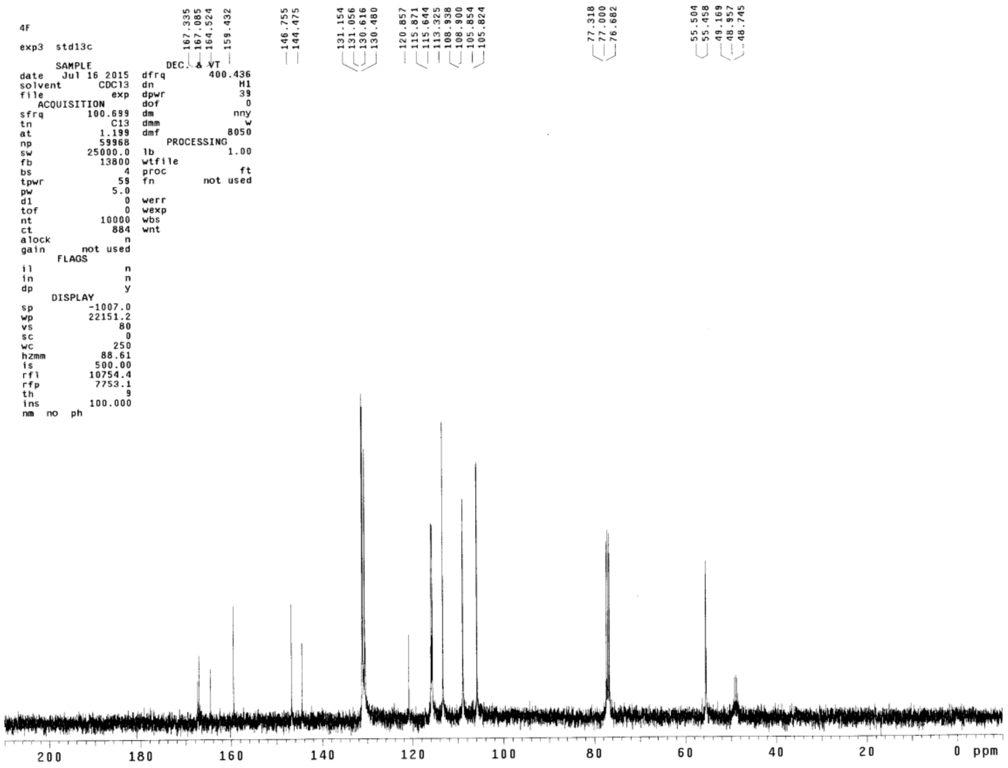

Fig. S14 The ^{13}C NMR Spectrum of Compound **5d** in $CDCl_3$ (100MHz)

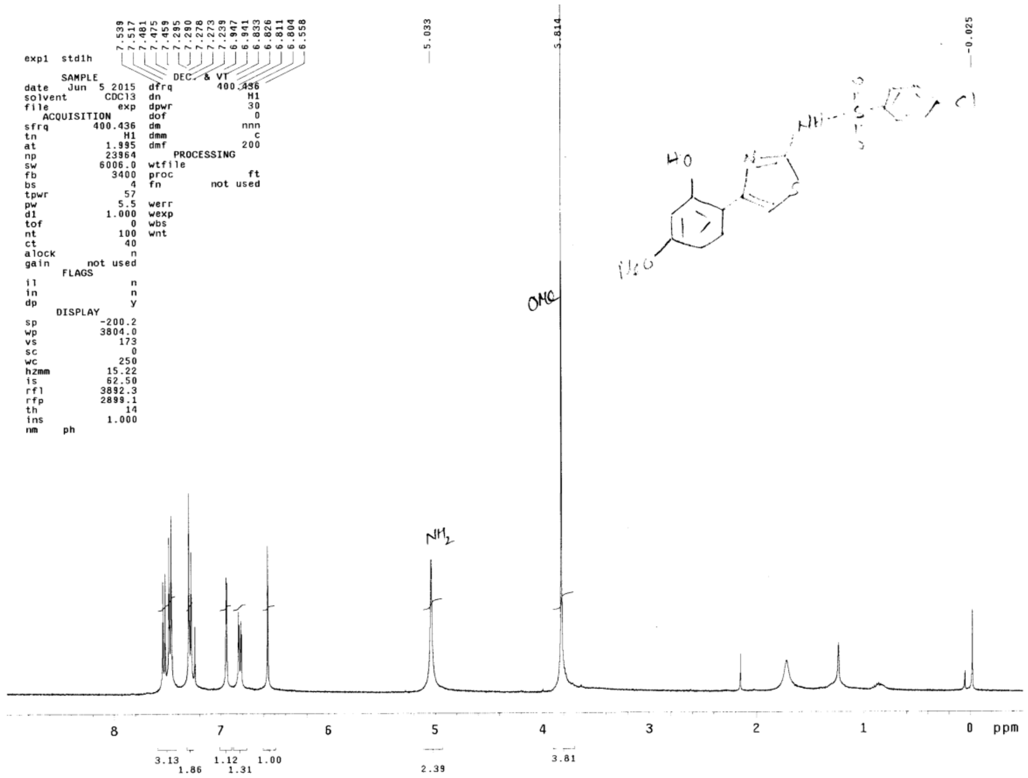

Fig. S15 The ^{1}H NMR Spectrum of Compound **5e** in $CDCl_3$ (400 MHz)

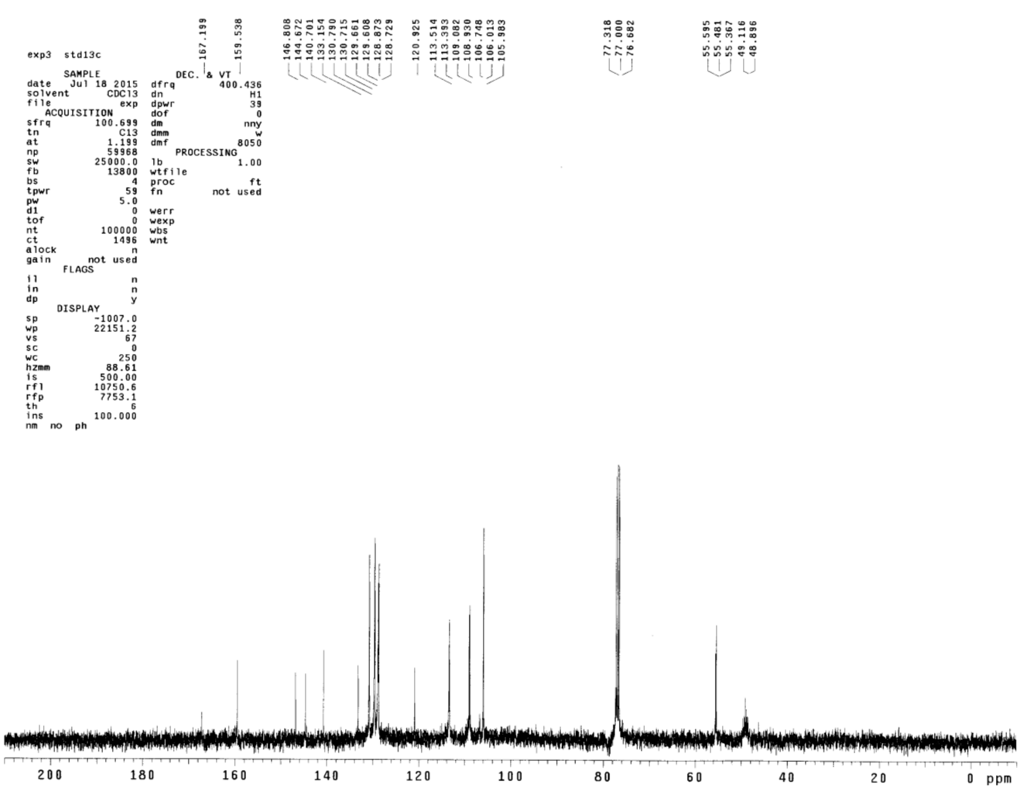

Fig. S16 The ^{13}C NMR Spectrum of Compound **5e** in $CDCl_3$ (100MHz)

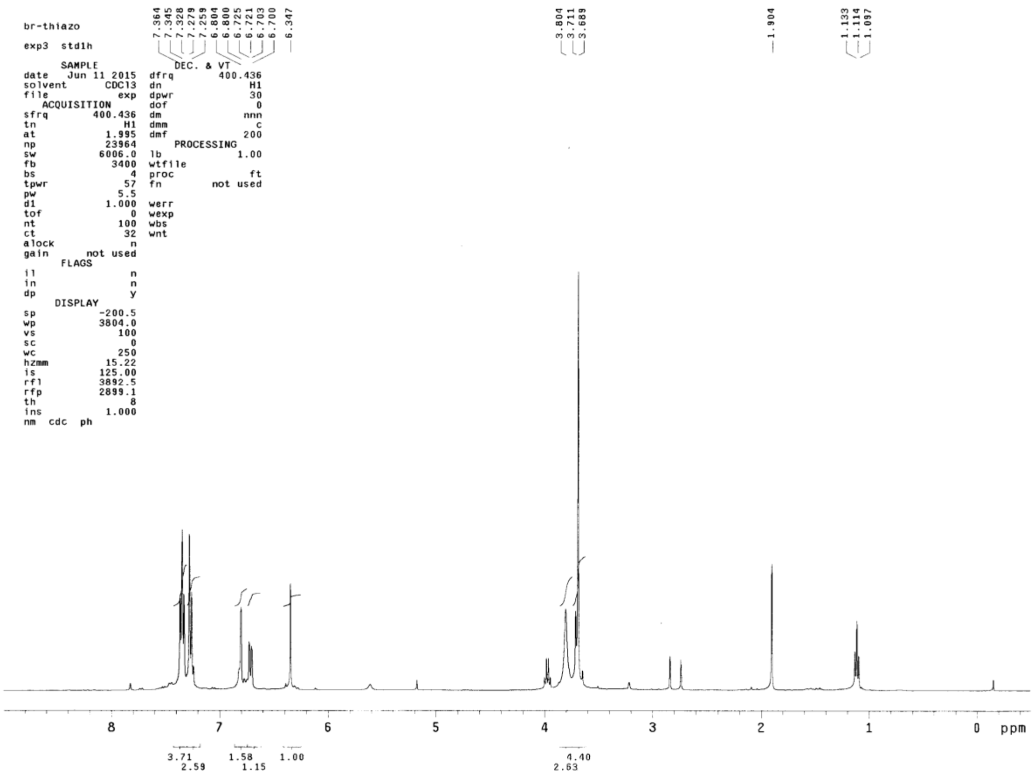

Fig. S17 The ^{1}H NMR Spectrum of Compound **5f** in $CDCl_3$ (400 MHz)

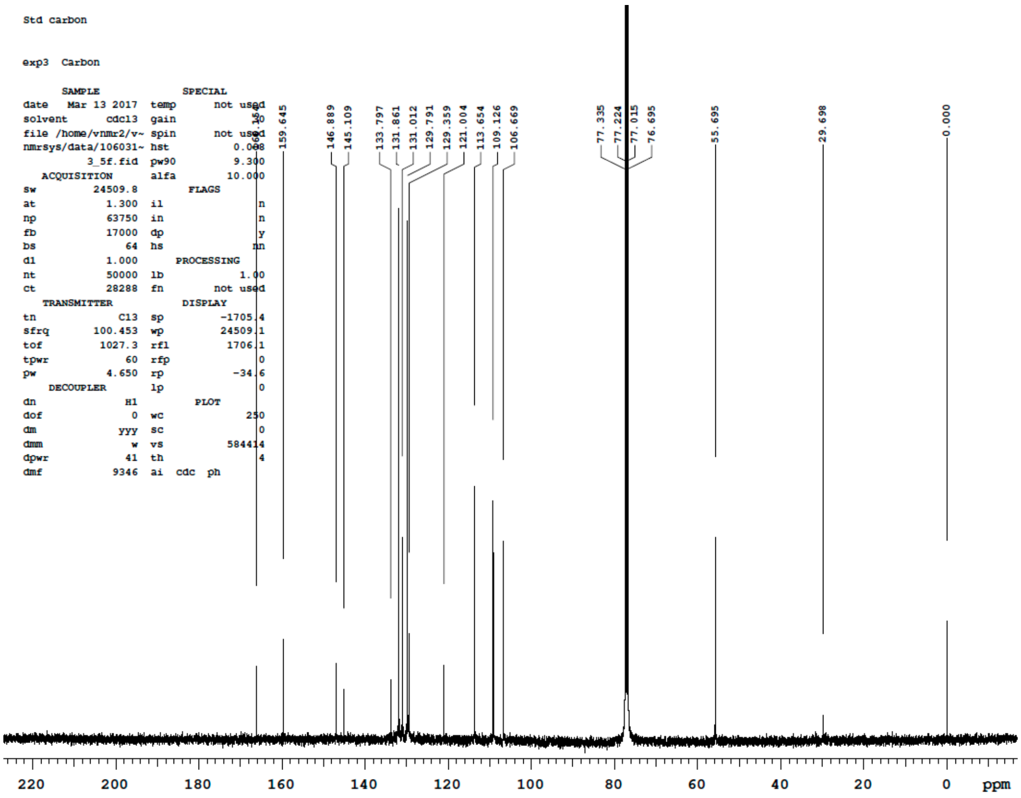

Fig. S18 The ^{13}C NMR Spectrum of Compound **5f** in $CDCl_3$ (100MHz)

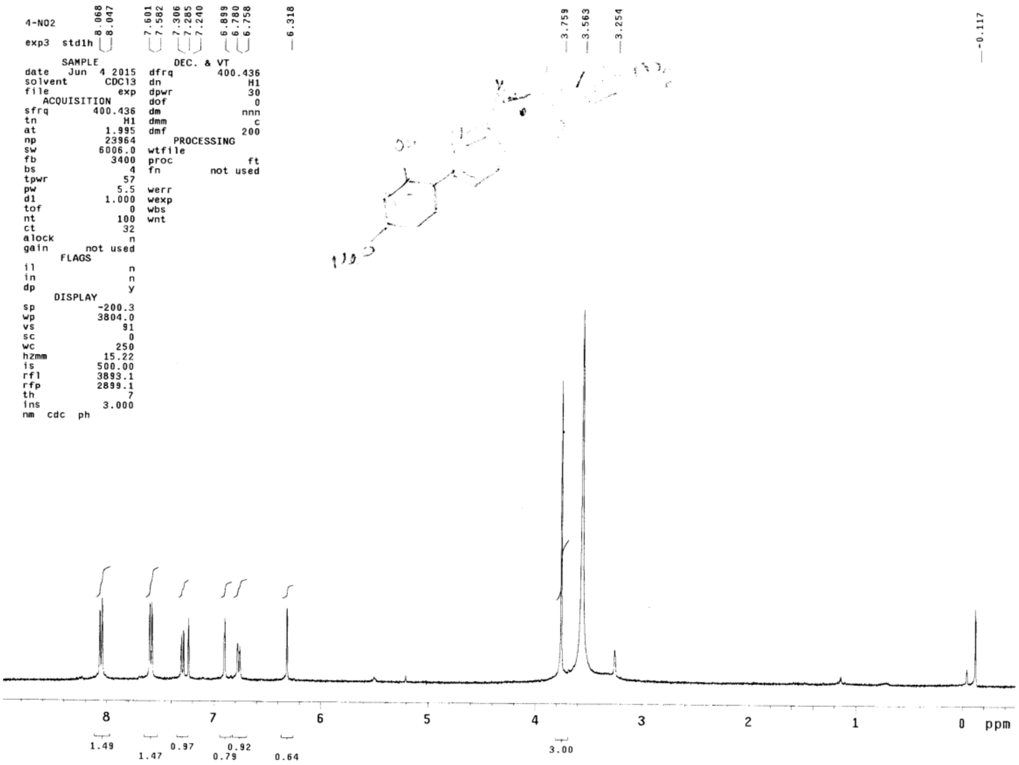

Fig. S19 The ^{1}H NMR Spectrum of Compound **5g** in $CDCl_3$ (400 MHz)

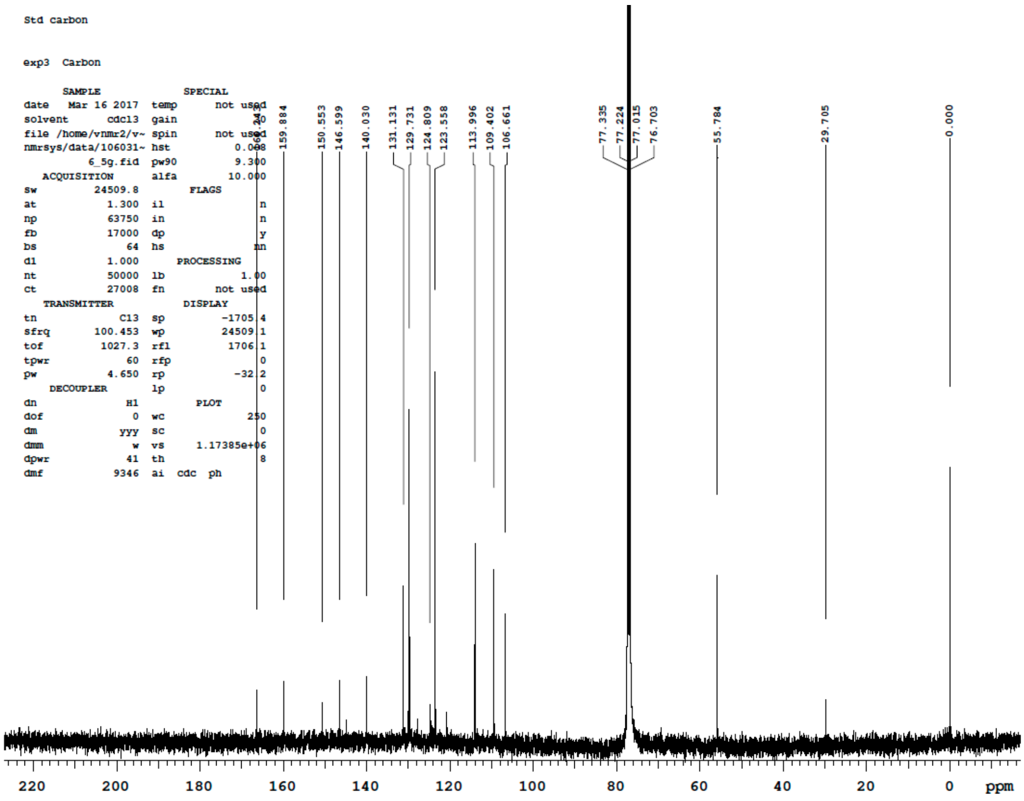

Fig. S20 The ^{13}C NMR Spectrum of Compound **5g** in $CDCl_3$ (100MHz)
